# Microtubule depolymerization contributes to spontaneous neurotransmitter release in vitro

**DOI:** 10.1038/s42003-023-04779-1

**Published:** 2023-05-05

**Authors:** Cecilia D. Velasco, Rachel Santarella-Mellwig, Martin Schorb, Li Gao, Oliver Thorn-Seshold, Artur Llobet

**Affiliations:** 1 Laboratory of Neurobiology, Department of Pathology and Experimental Therapy, Institute of Neurosciences, University of Barcelona, 08907 L’Hospitalet de Llobregat, Barcelona, Spain; 2 Bellvitge Biomedical Research Institute (IDIBELL), 08907 L’Hospitalet de Llobregat, Barcelona, Spain; 3 Electron Microscopy Core Facility, European Molecular Biology Laboratory (EMBL), Meyerhofstrasse 1, 69117 Heidelberg, Germany; 4 Department of Pharmacy, Ludwig-Maximilians University of Munich, Munich, 81377 Germany

**Keywords:** Cellular neuroscience, Synaptic transmission

## Abstract

Microtubules are key to multiple neuronal functions involving the transport of organelles, however, their relationship to neurotransmitter release is still unresolved. Here, we show that microtubules present in the presynaptic compartment of cholinergic autaptic synapses are dynamic. To investigate how the balance between microtubule growth and shrinkage affects neurotransmission we induced synchronous microtubule depolymerization by photoactivation of the chemical inhibitor SBTub3. The consequence was an increase in spontaneous neurotransmitter release. An analogous effect was obtained by dialyzing the cytosol with Kif18A, a plus-end-directed kinesin with microtubule depolymerizing activity. Kif18A also inhibited the refilling of the readily releasable pool of synaptic vesicles during high frequency stimulation. The action of Kif18A was associated to one order of magnitude increases in the numbers of exo-endocytic pits and endosomes present in the presynaptic terminal. An enhancement of spontaneous neurotransmitter release was also observed when neurons were dialyzed with stathmin-1, a protein with a widespread presence in the nervous system that induces microtubule depolymerization. Taken together, these results support that microtubules restrict spontaneous neurotransmitter release as well as promote the replenishment of the readily releasable pool of synaptic vesicles.

## Abstract (web summary)

Microtubules restrict spontaneous neurotransmitter release and favour the replenishment of the readily releasable pool of synaptic vesicles in cholinergic autapses.

## Introduction

Microtubules participate in many fundamental biological processes, including intracellular transport and cell division. Neurons, which show an extremely polarized morphology, use microtubules to establish efficient communication among their compartments. This is a key role to synapses, since they are located distally from the cell body and rely on the fast transport of organelles to correctly maintain their functions^1^. However, microtubules do not only contribute to synapse homeostasis. The balance between their assembly and disassembly provides a dynamism used to transiently reach small synaptic compartments and carry out specific functions. For example, the polymerization of microtubules in response to neuronal activity contributes to the delivery and removal of specific cargo in dendritic spines^2,3^. But despite the well-established relevance of microtubule dynamics to postsynaptic properties, there are substantial knowledge gaps about their importance at the presynaptic level.

Microtubules form a marginal band in the presynaptic compartment that concentrates mitochondria^4,5^. This structural role complements their ability to establish interactions with synaptic vesicle pools and it is illustrated by their participation in the delivery of synaptic vesicle precursors^6^ or the maintenance of neurotransmitter release during high-frequency stimulation^7^. The capacity of microtubules to directly interact with synaptic vesicle proteins such as synapsin^8^ or synaptotagmin^9^ may be part of the molecular mechanisms regulating their role in the presynaptic compartment. The contribution of microtubule dynamic instability has, however, not been considered in functional observations.

Neurons contain numerous stable microtubules^10^ that co-exist with a dynamic population present along axons and boutons^11,12^ whose role must be accounted for. Since the early observations of Gray^13^, electron microscopy studies show that presynaptic microtubules are non-uniformly distributed within the presynaptic terminal; they emanate from the axon and can be virtually absent or appear closely located to active zones. Such differences can reflect a balance between polymerization and depolymerization that is supported by the real-time visualization of microtubule growth in axon terminals forming neuromuscular junctions^14^.

Whether the equilibrium between microtubule shortening and growth affects their relationships with synaptic vesicles is yet unknown. This question has remained elusive mainly due to multiple experimental constraints, such as the small size and limited accessibility of presynaptic terminals or, the lack of probes capable of manipulating microtubule dynamics on a fast temporal scale. In this study, we take advantage of the experimental possibilities offered by neurons forming autaptic synapses and, of photoactivable microtubule inhibitors that permit rapidly resolved studies. Together with experiments using correlative electrophysiology and electron microscopy, our work reveals a direct role of microtubules in neurotransmission, supporting that the normal balance between polymerization and depolymerization is important to restrict spontaneous neurotransmitter release and the refilling of synaptic vesicle pools.

## Results

### Microtubule plus ends transiently invade the presynaptic compartment

**Fig. 1:**
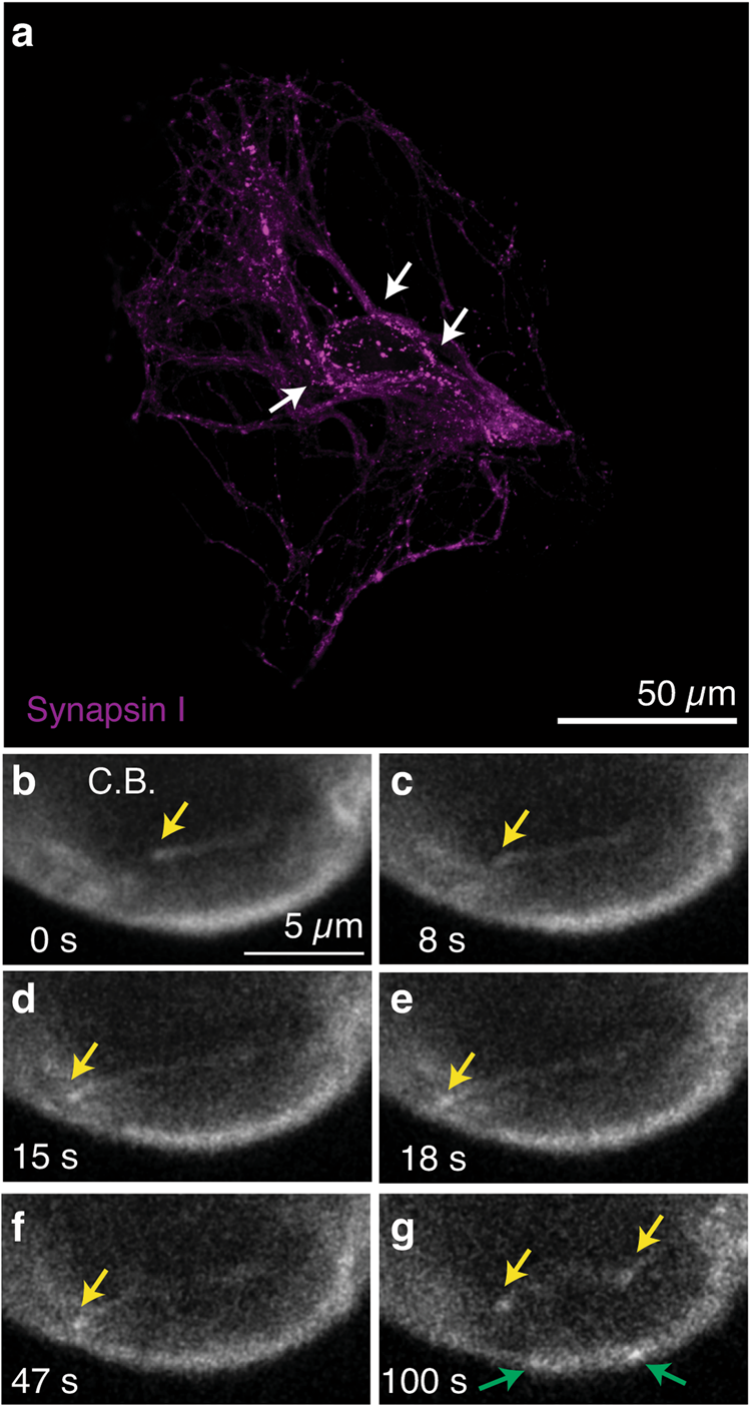
Movement of EB3-tdTomato comets in a cholinergic autaptic culture. **a** Expression of synapsin I in a cholinergic single-cell microculture (SCM). Arrows indicate the location of putative axosomatic autapses. **b**–**g** Movement of EB3-tdTomato comets in the periphery of the cell body (C.B.) of an autaptic neuron. Images **b**–**e** show the movement of a single comet (yellow arrows) at the indicated times. New comets appear along a neurite (yellow arrows in **f** and **g**) and show similar traits. Comets transiently appear in the perisomatic region (green arrows in **g**). Data are extracted from Supplementary Movie 1.

Single-cell microcultures (SCMs) are established from rat superior cervical ganglion neurons and allow the investigation of the function of a population of autaptic contacts in the absence of glia^15^. Neuronal growth is constrained to a 200–300 μm diameter collagen microdot that forces the axon to establish reciprocal synapses in a monosynaptic circuit. Although many axodendritic synapses are present within the microculture, axosomatic synapses are the major contributors of recorded currents due to the cable filtering properties of neurites^16^. Potential axosomatic synapses are easy to identify by staining with a synaptic marker^17^ and appear as discrete puncta in the periphery of the cell body (Fig. 1a). To monitor microtubule plus end dynamics, neurons were infected with lentiviruses to induce the expression of EB3-tdTomato, a construct based on a +TIP protein that is present in growing microtubules^18^. EB3 comets were obvious along neurites and displayed hallmarks of the dynamics observed in cultured neurons^19,20^, characterized by a sudden appearance followed by a movement in a preferred direction (Supplementary Movie 1). Velocity was typically in the range of 0.2 to 0.3 μm·s^−1^ and traveled distances were found between 3 and 7 μm (Fig. 1b–e). Imaging at the level of the cell body showed that microtubule growth was initiated or terminated in defined perisomatic locations (Fig. 1f, g). Considering the high density of axosomatic synapses, this observation could reflect the presence of boutons acting as sites of microtubule nucleation^12^ or positioning of plus ends^21^, respectively.

**Fig. 2:**
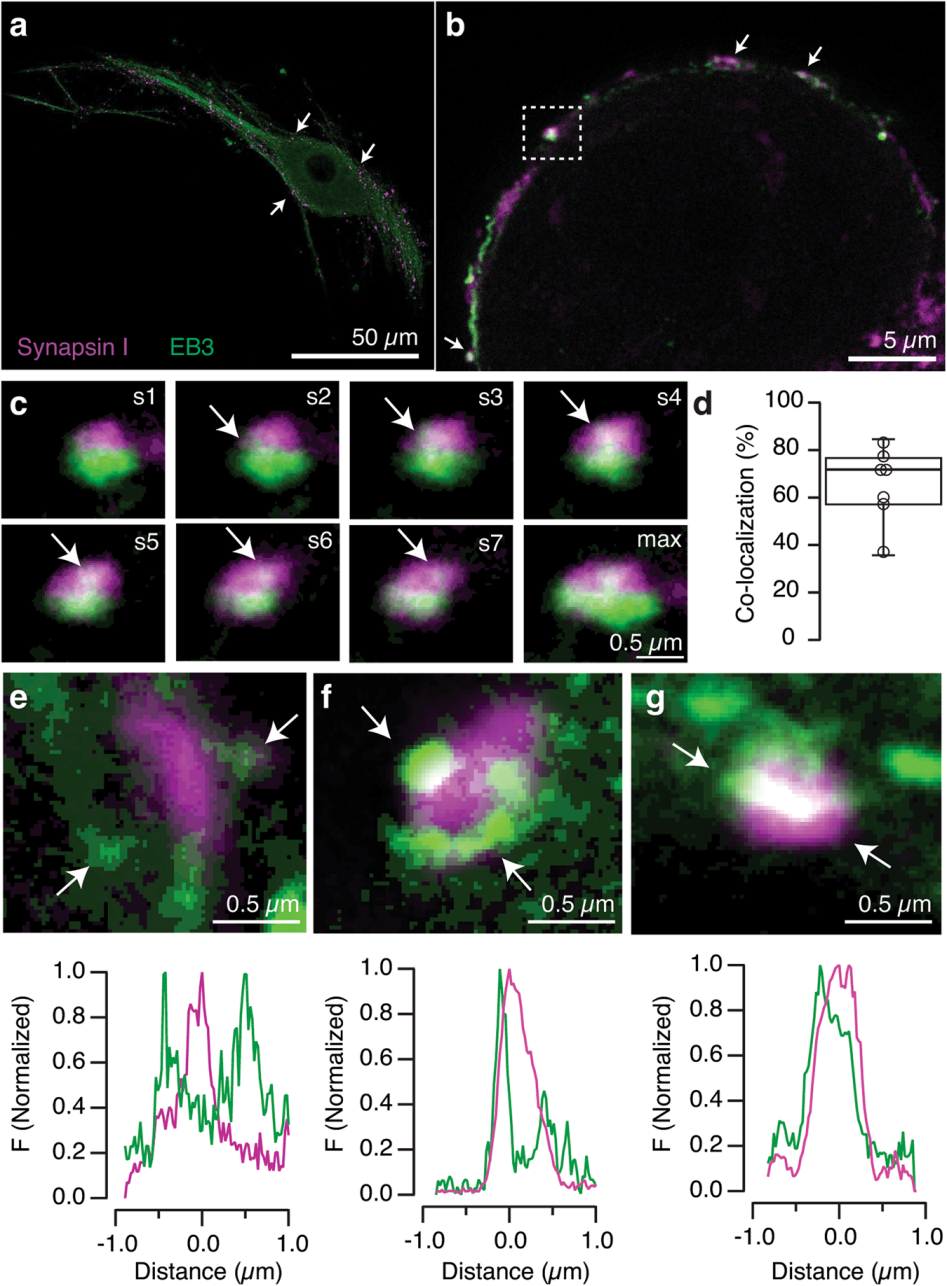
STED microscopy reveals the invasion of synapsin clusters by EB3 comets. **a** Maximum intensity projection of a Single Cell Microculture (SCM) stained for synapsin I and EB3. Arrows indicate the location of the cell body. **b** Putative axosomatic synapses labeled with synapsin I are also stained by EB3 (arrows). **c** Consecutive optical sections (s1–s7) of the cluster of synaptic vesicles boxed in (**b**) illustrate the invasion by EB3 comets. Notice that both labels are partially segregated and co-localize in a central patch (arrows) that becomes evident in the maximum intensity projection (max). **d** Box plot showing the median (horizontal line), 25 to 75% quartiles (boxes), and ranges (whiskers) of the co-localization found between EB3 and synapsin I in the axosomatic contacts of 7 different SCMs. **e**–**g** Images show examples of the relationships established between EB3 comets and axosomatic synapsin I clusters. Line profiles drawn between arrows indicated in each image display the absence (**e**), partial (**f**), or complete (**g**) co-localization of EB3 with synapsin I.

The interpretation of EB3-tdTomato imaging in autaptic cultures is complex since the same neuron defines the presynaptic and the postsynaptic element. Moreover, the ∼1 μm diameter of axon terminals, makes it impossible to discriminate by conventional optical microscopy whether microtubule plus ends co-localizing with synaptic vesicle markers reflect locations inside or outside of the synaptic bouton. We overcame this limitation by visualizing EB3 expression with STED microscopy. An uneven staining was evident along the neuritic tree of SCMs and co-localized with synapsin I, a protein present in the synaptic vesicle membrane (Fig. 2a). Puncta located in the perimeter of the cell body were considered as axosomatic synapses if synapsin I stain appeared in 5–7 consecutive optical sections (0.8–1.1 μm) and had a ∼1 μm diameter (Fig. 2b, c). EB3 showed a patchy appearance, consistent with the formation of comets at axonal and dendritic locations. Although many synaptic vesicle clusters were surrounded by microtubule plus ends, co-localization of both signals was not always observed. EB3 comets formed protrusions that appeared as an elongated profile spanning an average of 6 optical sections and were found invading 64 ± 6% of synapsin I puncta (mean ± s.e.m., *n* = 215, Fig. 2d, Supplementary Data 1). These data are reminiscent of the calyx of Held, where partial co-localization of synaptic vesicles and microtubules is also observed^7^.

The absence or presence of dynamic microtubule plus ends in synapsin clusters could be associated to two different populations of axon terminals but, the analysis of EB3 fluorescence signals rejected a binary distribution. The degree of overlay of EB3 and synapsin I was variable, so we classified the distribution of both markers as absent (Fig. 2e), partial (Fig. 2f), or complete co-localization (Fig. 2g). The first situation, defined by the lack of overlap, indicates the presence of microtubule plus ends in axonal or dendritic regions excluded from the presynaptic compartment. The second and third situations are consistent with the establishment of a transient interaction between EB3 comets and synaptic vesicles. Microtubule plus ends rapidly shorten in the micrometer range upon catastrophe and regrow slowly until experiencing a new rapid depolymerization process^22^. The images shown in Fig. 2e–g are consistent with the existence of a continuous process where microtubule plus ends enter and exit the presynaptic compartment and establish a transient interaction with the synaptic vesicle cluster.

### Hallmarks of microtubule organization in a small presynaptic terminal

**Fig. 3:**
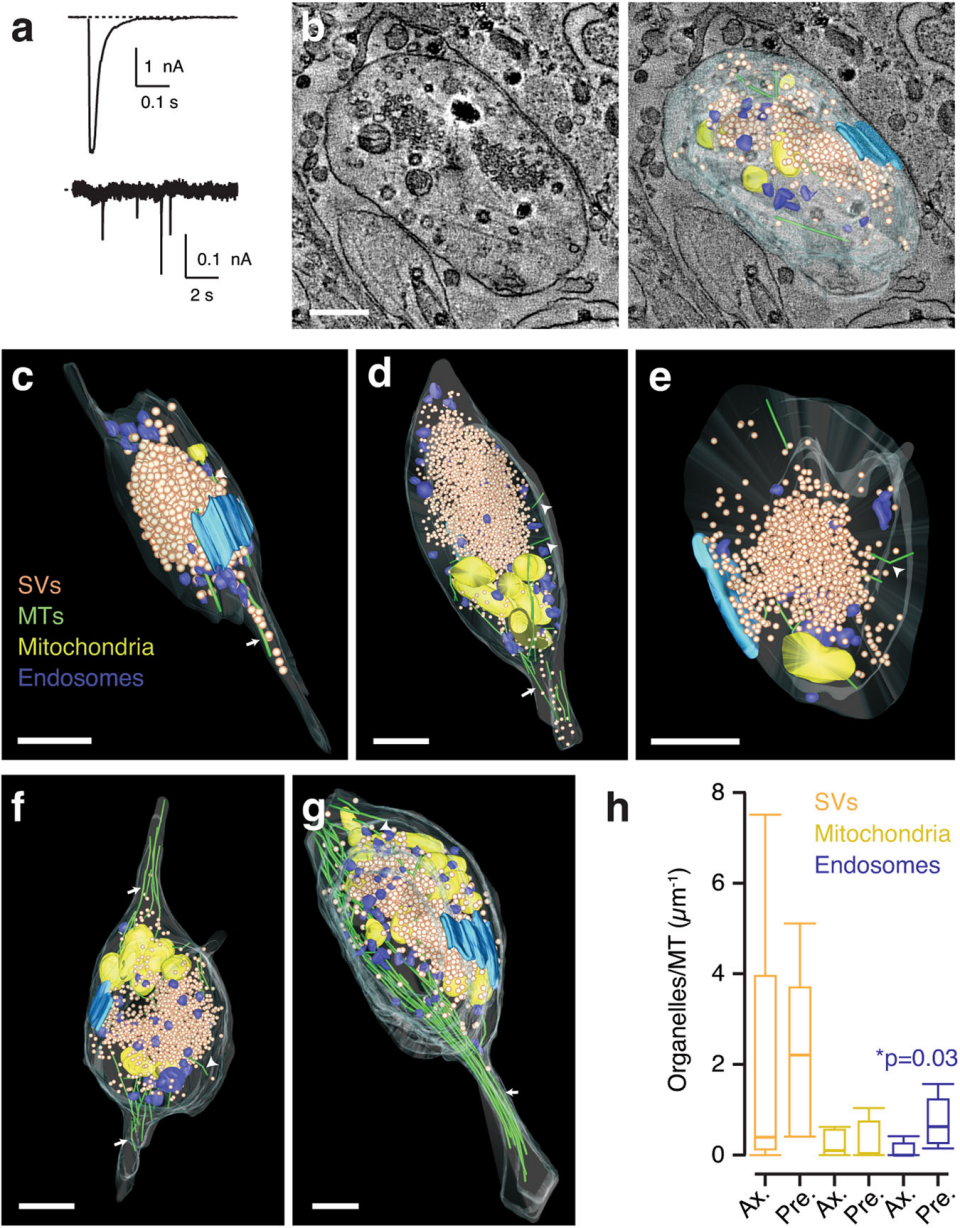
Correlative electrophysiology and serial electron tomography. **a** A neuron with representative evoked and spontaneous neurotransmitter release was fixed and prepared for serial electron tomography. **b** The structures of five presynaptic terminals establishing synapses on the cell body (**c**–**e**) or proximal dendrites (**f**, **g**) were obtained by serial electron tomography and subsequently segmented to track the position of synaptic vesicles (orange), microtubules (green), mitochondria (yellow), endosomes (blue) and active zones (cyan). **h** Box plots showing the median (horizontal line), 25 to 75% quartiles (boxes), and ranges (whiskers) of the frequency of organelles found along microtubules connected to the axon (Ax., arrows) and along microtubules entirely found within the presynaptic compartment (Pre., arrowheads). Differences were established using the Mann–Whitney test (*n* = 5). Scale bars indicate 500 nm.

To gain insight into how microtubules were distributed within an axon terminal we carried out experiments of correlative electrophysiology and automated serial electron tomography. An autaptic neuron showing the stereotyped neurotransmission of SCMs, which is characterized by miniature excitatory postsynaptic currents (mEPSCs) occurring at ∼0.1 Hz and EPSC amplitudes ranging from 2 to 4 nA (Fig. 3a), was fixed and prepared for electron microscopy (see “Methods” for details). In the volumes obtained by tomography, it was possible to reconstruct 2 axosomatic synapses and 3 axodendritic synapses proximal to the cell body. The method is illustrated in Fig. 3b, Supplementary Movies 2 and 3. Synaptic contacts found in distant locations to the cell body were not investigated, since their contribution to recorded neurotransmission was minimal.

The structure of presynaptic terminals was remarkably similar and was characterized by the presence of a single active zone and a central cytoplasmic pool of synaptic vesicles (Fig. 3c–g, Supplementary Movies 4–8). Active zones contained 8 ± 1 (mean ± s.e.m., *n* = 5) synaptic vesicles, in agreement with previous estimates^23^. The cytoplasmic pool contained 1403 ± 274 vesicles and the mean volume of axon terminals was 1.12 ± 0.4 μm^3^ (mean ± s.e.m., *n* = 5), both about one order of magnitude greater than boutons of hippocampal and cortical neurons^24^. Mitochondria adopted a peripheral distribution and were associated to a marginal band of microtubules, thus resembling the organization present in retinal bipolar cell terminals^4^. Terminal size, the density of synaptic vesicles, the number of active zones, and the distribution of mitochondria were comparable among all terminals investigated, thus evidencing that the structural features of presynaptic terminals investigated (Fig. 3c–g) were representative of cholinergic autaptic synapses established by a given neuron. Such reproducible morphological hallmarks contrasted with the variable length and number of microtubules invading axon terminals. It was possible to find presynaptic terminals containing a dense network of long microtubules (Fig. 3f, g) as well as others presenting a scarce distribution (Fig. 3c–e). These differences were consistent with ongoing dynamic instability and agreed with the findings obtained by imaging of EB3-tdTomato or STED microscopy (Figs. 1 and 2).

There were 16 ± 3 (mean ± s.e.m., *n* = 5) synaptic vesicles attached to microtubules forming the presynaptic network, which represented about 1.4% of the total vesicle pool. Some presynaptic microtubules extended to the axon (Fig. 3c, d, f, g, arrows), while others showed their entire length restricted to the presynaptic compartment (Fig. 3c–g, arrowheads). A group of microtubules originating in the axon showed a branched appearance, hence it is possible that some of those identified only in the presynaptic compartment were microtubules growing out from nucleation points (Supplementary Fig. 1). The microtubules that were observed in close contact to the cytoplasmic vesicle pool did not get inserted into the vesicle cluster. The capacity to tether vesicles and mitochondria was similar for all microtubules identified, e.g., on average, those that extended to the axon bound a vesicle every 585 nm while those restricted to the axonal terminal did so every 480 nm (Fig. 3h, Supplementary Data 2). The vicinity of endosomes to the synaptic vesicle cluster favored their interaction with microtubules whose entire length was restricted to the presynaptic terminal. On average they bound an endosome every 1382 nm, whilst microtubules extending to the axon did it every 9696 nm.

### Microtubule depolymerization selectively increases spontaneous neurotransmitter release

To gain knowledge on the consequences of microtubule catastrophes in the synaptic bouton, we induced synchronous microtubule depolymerization. It can be achieved within minutes^25^ using microtubule destabilizing chemical reagents such as colchicine and combretastatin A-4 (CA-4), however, the inability to apply these agents with spatiotemporal control precludes their specific targeting to synapses. Better suited to our needs are the photoswitchable analogs of CA-4, e.g., photostatins (PSTs), that allow the suppression of microtubule polymerization with time- and location-targeted precision, directed by light^26^. PSTs were the first photoswitchable microtubule inhibitors to be developed. They rapidly enter cells and can then be used to switch microtubule polymerization off and on by alternating illuminations with UV light (*E* → *Z* isomerization, i.e., bioactivity switch-on) and green light (*Z* → *E* isomerization, i.e., bioactivity switch-off), with single-cell spatial precision. However, PSTs have drawbacks including unclear metabolic stability, limited compatibility with GFP/RFP imaging, and only moderate potency, which restrict their experimental possibilities. We thus used SBTub3, a newer photoswitchable depolymerizer with excellent GFP/RFP compatibility and metabolic stability, whose unidirectional *E* → *Z* photoactivation with UV light also reduces the number and lifetime of EB3 comets in neurites^27^.

**Fig. 4:**
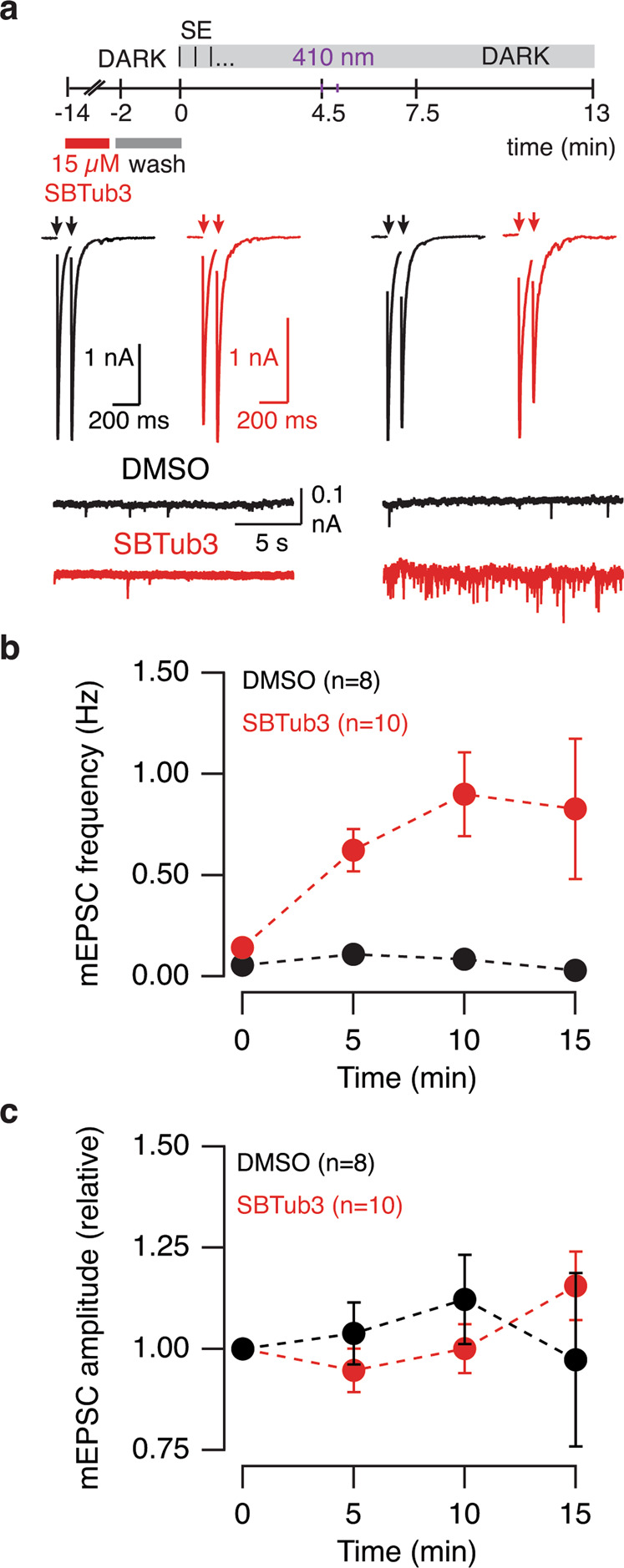
*E* → *Z* Photoactivation of SBTub3 selectively increases spontaneous neurotransmitter release. **a** Overview of the experimental protocol. Spontaneous and evoked neurotransmission were evaluated alternately in single-cell microcultures (SCMs) incubated with 15 μM *E-*SBTub3 (gray box). Miniature excitatory postsynaptic currents (mEPSCs) were recorded over a 30 s period (S) followed by the application of two stimuli (arrows) at a 100 ms interval (E). Photoactivation consisted in the delivery of two light pulses (3 s each, 410 nm) in a 30 s interval. Evoked neurotransmission was not affected by *E* → *Z* photoactivation of SBTub3, whereas the frequency of mEPSCs increased. **b**, **c** Photoactivation of SBTub3 increased the frequency of mEPSCs but their amplitude was not modified and was comparable to values found in SCMs incubated with vehicle (0.15% DMSO). Dots indicate mean ± s.e.m.

Spontaneous and evoked neurotransmission were monitored in SCMs incubated with 15 μM *E-*SBTub3. To be included in the analysis, neurons had to show a stable holding current below 0.1 nA and a mEPSC frequency below 0.1 Hz over a baseline period of 4.5 min. Illumination with 2 light pulses of 410 nm at an interval of 30 s (*E* → *Z* photoactivation) enhanced spontaneous neurotransmitter release (Fig. 4a). Light-induced changes led to an increase in the frequency but not the amplitude of mEPSCs (Fig. 4b, c, Supplementary Data 3). These results were consistent with the increase of more than one order of magnitude in spontaneous neurotransmitter release observed in SCMs incubated with CA-4 (Supplementary Fig. 2). Neither synaptic strength nor paired-pulse plasticity were modified by *E* → *Z* photoactivation of SBTub3, suggesting that presynaptic terminal properties were differently affected by the synchronous shortening of microtubules.

**Fig. 5:**
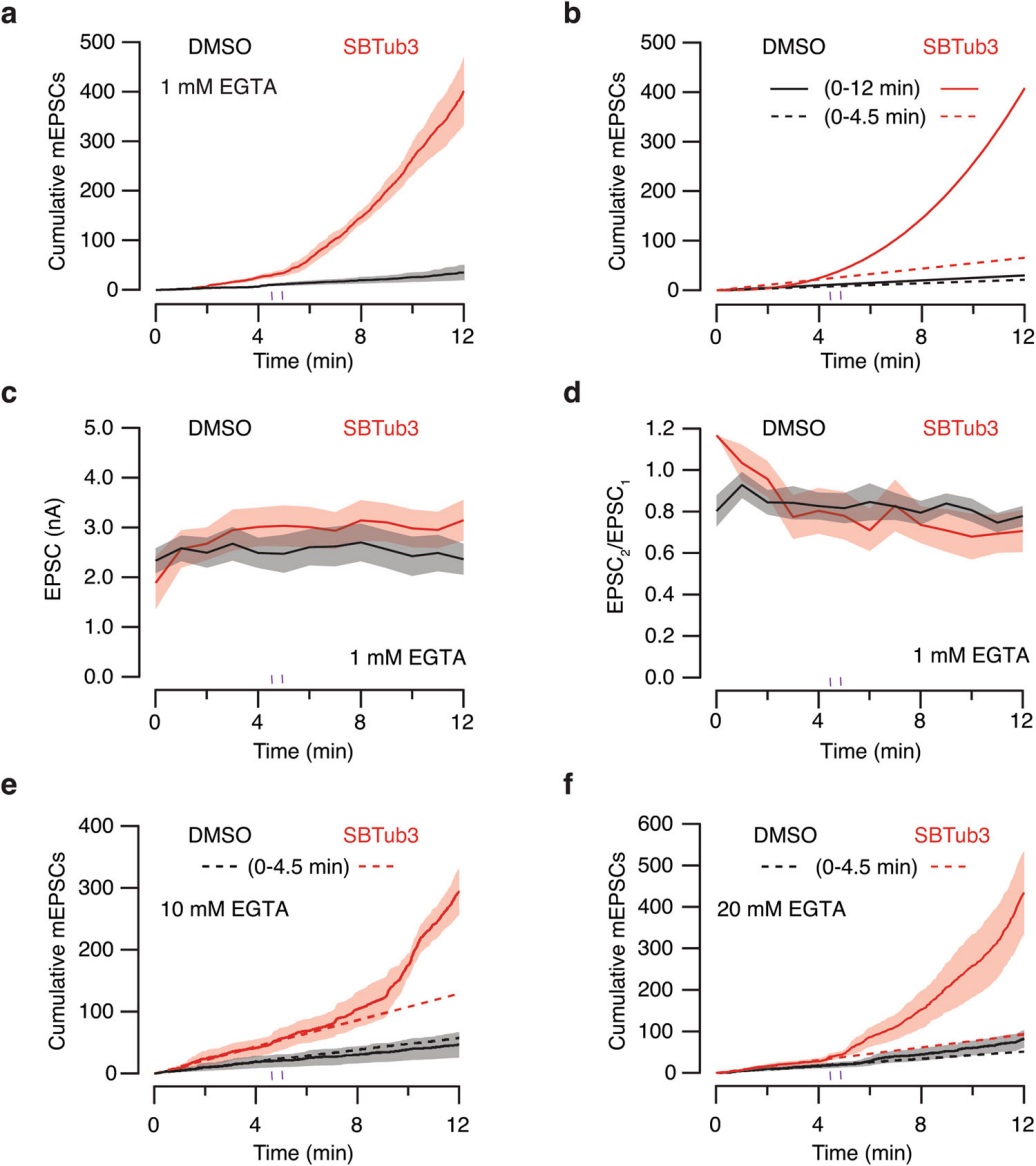
Spontaneous neurotransmitter release increases exponentially after photoactivation of SBTub3. **a** Cumulative increase in miniature excitatory postsynaptic currents (mEPSCs) over experimental time in single-cell microcultures (SCMs) incubated with SBTub3 (*n* = 9) or vehicle (0.15% DMSO, *n* = 13). Neurons were dialyzed with standard internal solution containing 1 mM EGTA. Lines and shadowed areas indicate mean ± s.e.m. **b** mEPSCs occurred at a linear rate before *E* → *Z* photoactivation of SBTub3 (dotted lines). The linear increase in spontaneous neurotransmitter release persisted after the delivery of two light pulses (3 s each, 410 nm, 30 s interval) in neurons incubated with DMSO but changed to exponential after photoactivation of SBTub3 (solid line). **c**, **d** Synaptic strength measured as the amplitude of EPSCs (**c**) and paired-pulse ratio (**d**) were comparable in neurons incubated with SBTub3 (*n* = 9) or 0.15% DMSO (*n* = 13). Lines and shadowed areas indicate mean ± s.e.m. **e**, **f** *E* → *Z* photoactivation of SBTub3 changed the cumulative number of mEPSCs as a function time from linear to exponential when the internal solution contained 10 mM (*n* = 4) or 20 mM EGTA (*n* = 7), whereas it increased linearly when neurons were incubated with 0.15% DMSO (10 mM EGTA, *n* = 4; 20 mM EGTA, *n* = 10). Lines and shadowed areas indicate mean ± s.e.m.

The time course of changes in the frequency of spontaneous events was evaluated by plotting the cumulative number of mEPSCs as a function of experimental time (Fig. 5a, Supplementary Data 4). Neurons incubated with 0.15% DMSO showed a linear increase in spontaneous neurotransmitter release that occurred at a rate of 1.8 mEPSCs min^−1^ before light exposure (*n* = 13, Fig. 5b). Illumination with 410 nm light did not affect the overall rate of mEPSCs, which occurred at 2.5 mEPSCs·min^−1^ during the whole period investigated. In contrast, neurons incubated with inactive *E-*SBTub3 had a linear increase of 5.4 mEPSCs·min^−1^ measured before 410 nm illumination. Upon exposure to 410 nm light pulses (*E* → *Z*-SBTub3) this linear growth changed to exponential. Data were fit empirically to a power function where the total number of mEPSCs observed at a given experimental time (t) followed the relationship 0.7t^2.75^ (*n* = 9, Fig. 5b). The change from a linear to an exponential increase in mEPSC rate indicates that microtubule depolymerization promoted spontaneous neurotransmitter release. This observation contrasts to the lack of effect on evoked neurotransmission since the amplitude of EPSCs remained constant either in the absence or presence of *Z-*SBTub3 (after illumination; Fig. 5c, Supplementary Data 4). Paired-pulsed plasticity was also unaltered in both experimental groups (Fig. 5d, Supplementary Data 4).

To be considered as spontaneous, neurotransmitter release must occur in the presence of basal cytosolic calcium concentration. We therefore wished to test whether the free calcium concentration of the cytosol had been basal, or if the delivery of paired-pulse stimuli during the experimental protocol (Fig. 4a), or light-damaging effects, could have increased it. Since sub-micromolar increases in presynaptic calcium levels can support neurotransmitter release^28^ we sought to clamp the cytoplasmic calcium concentration by dialyzing neurons with intracellular solutions containing 10 mM and 20 mM EGTA (Fig. 5e, f, Supplementary Data 4). The 10-fold and 20-fold increase in calcium buffering did not alter the action of *Z-*SBTub3: the increase in the rate of mEPSCs was comparable to neurons dialyzed with 1 mM EGTA. The total number of mEPSCs observed when the cytosol contained 10 mM and 20 mM EGTA was described as a function of time (t) by the power functions 1.4t^2.1^ and 1.2t^2.35^, respectively. Synaptic strength also remained unaltered when the cytosol contained high EGTA concentrations (Supplementary Fig. 3), thus confirming that *E* → *Z* photoactivation of SBTub3 increased spontaneous neurotransmitter release.

### Kif18A increases spontaneous neurotransmitter release in a concentration-dependent manner

**Fig. 6:**
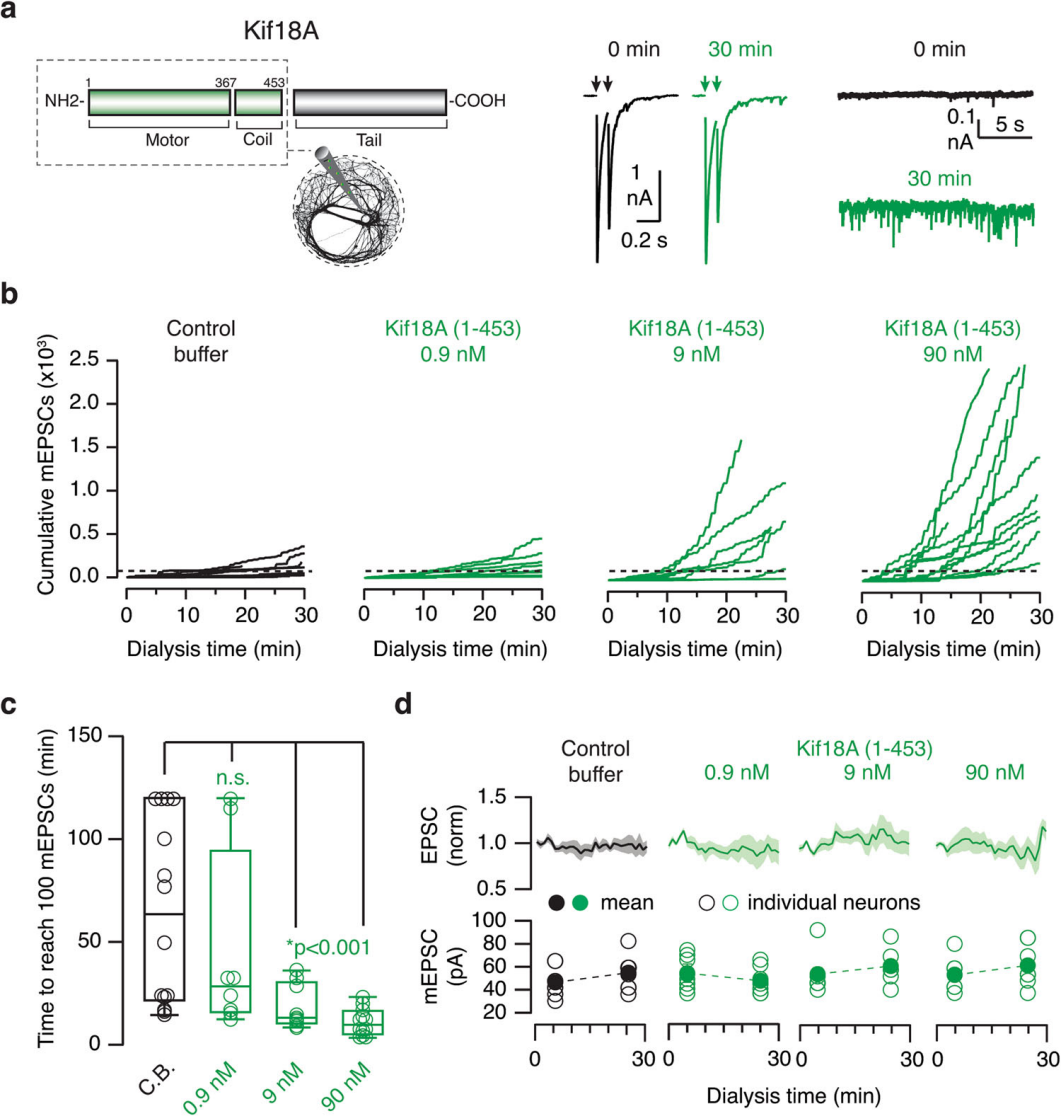
Kif18A selectively increases spontaneous neurotransmitter release. **a** Neurons were recorded in the whole-cell configuration of the patch-clamp technique. The internal solution contained 0.9 nM (*n* = 8), 9 nM (*n* = 8), 90 nM (*n* = 12) of recombinant Kif18A(1–453) or control buffer (C.B., *n* = 14). Excitatory postsynaptic currents (EPSCs, arrows) and miniature EPSCs (mEPSCs) recorded in a neuron dialyzed with 90 nM Kif18A(1–453). **b** The increase in the cumulative number of mEPSCs as function of time was dependent on the concentration of Kif18A(1–453) in the patch pipette. Each trace shows the spontaneous activity of a single neuron. Dotted line indicates 100 mEPSCs. **c** Time required to observe 100 mEPSCs as a function of Kif18A(1–453) concentration. Box plot shows the median (horizontal line), 25 to 75% quartiles (boxes), and ranges (whiskers) of the experimental groups indicated. Statistical differences were evaluated using one-way ANOVA followed by Dunnett’s multiple comparisons test. **d** Neither EPSC nor mEPSC amplitude increased during dialysis of the cytosol with Kif18A(1–453). Line and shadowed areas indicate mean±s.e.m amplitude of EPSCs. The mean amplitude of mEPSCs at the beginning and end of experimental time is shown by two dots. Error bars indicate s.e.m. Circles show individual values.

Although the use of photoswitchable microtubule inhibitors showed a direct contribution of microtubule depolymerization to neurotransmitter release, there was the possibility that the results obtained were related to a toxic and not to a biologically relevant effect. We next sought to create a near physiological regulation by taking advantage of biological factors that modify the balance between microtubule growth and catastrophe^29^. An example is Kif18A, a type of kinesin-8 with microtubule depolymerase activity (Fig. 6a). It plays a key role during mitosis^30^, it is not expressed in the brain (https://portal.brain-map.org) and contains three domains: motor, coiled-coil, and C-terminal^31^. Neurons were dialyzed with Kif18A(1–453),  a fragment that contains the depolymerase activity and plus end directed motility^32^, aiming to achieve a selective depolymerization of microtubule plus ends The presence of Kif18A(1–453) in the cytosol, which mimicked overexpression, showed a comparable effect to that of *E* → *Z* photoactivation of SBTub3. mEPSC frequency increased in a concentration-dependent manner (Fig. 6b, c, Supplementary Data 5), and the average linear increase of 2.5 mEPSCs·min^−1^ found in control neurons accelerated when the intracellular solution contained >9 nM Kif18A(1–453). Like for SBTub3 *E* → *Z* photoactivation, the change was characterized by a shift from linear to exponential growth in mEPSCs rate. Since it is not possible to set a precise onset time for Kif18A(1–453) action due to the intrinsic variability of its diffusion into the cytosol, the quantification of changes in the frequency of spontaneous events was established by measuring the time required to generate 100 mEPSCs (t_100_, Fig. 6c, Supplementary Data 5). The value of t_100_ when neurons were dialyzed with 0.9 nM Kif18A(1–453) was of 55 ± 23 min (*n* = 8), which shortened to 18 ± 4 min (*n* = 8) and 11 ± 3 min (*n* = 12) in the presence of 9 nM and 90 nM Kif18A(1–453), respectively. The probability of observing a single mEPSC was therefore increased as a function of Kif18A(1–453) concentration. Evoked neurotransmission remained unaltered in the presence of Kif18A(1–453), thus resembling results obtained upon *E* → *Z* photoactivation of SBTub3 (Fig. 6d, Supplementary Data 5). The amplitude of mEPSCs was not modified either, evidencing that the number or composition of postsynaptic receptors was not affected by Kif18A(1–453). Altogether, these experiments demonstrate that acute depolymerization of microtubule plus ends by a biological factor selectively potentiates spontaneous neurotransmitter release.

### Acute depolymerization of microtubules affects synaptic vesicle cycling

**Fig. 7:**
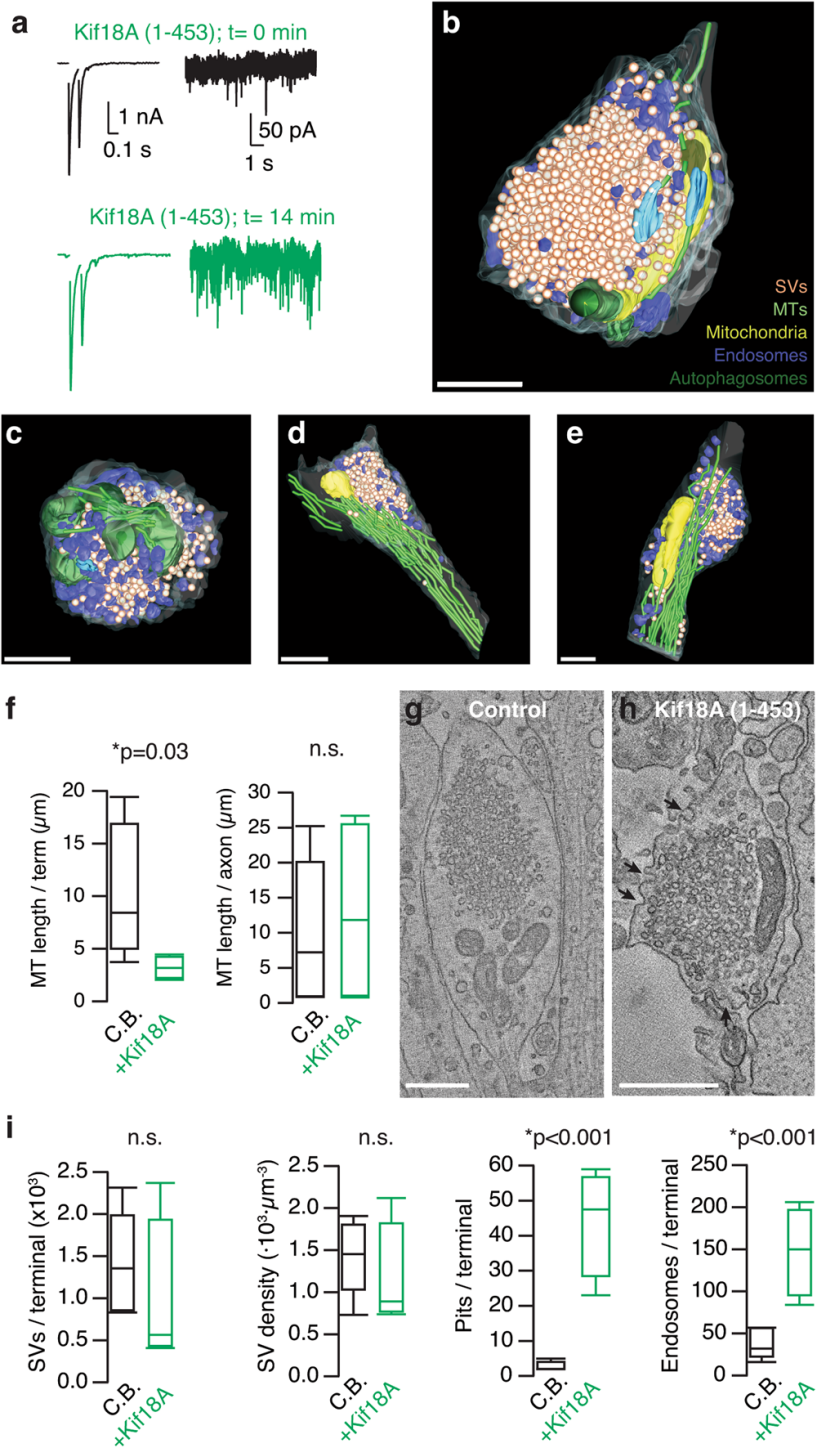
Kif18A disrupts microtubules and increases synaptic vesicle turnover. **a** A neuron with representative responses to dialysis with 90 nM Kif18A(1–453) was fixed and prepared for serial electron tomography. **b**–**e** The structures of four presynaptic terminals establishing synapses on the cell body (**b**, **c**) or proximal dendrites (**d**, **e**) were obtained by serial electron tomography and subsequently segmented to track the position of synaptic vesicles (orange), microtubules (green), mitochondria (yellow), endosomes (blue), autophagosomes (dark green), and active zones (cyan). **f** Box plots showing the median (horizontal line), 25 to 75% quartiles (boxes), and ranges (whiskers) of microtubule length found in the presynaptic terminal and the portion of axon analyzed. **g**, **h** A hallmark of Kif18A(1–453) action is the increase in the number of exo-endocytic pits (arrows) found in the presynaptic terminal membrane. **i** Box plots showing the median (horizontal line), 25 to 75% quartiles (boxes), and ranges (whiskers) of the experimental groups indicated (SV: synaptic vesicle). Statistical differences were established by comparing data obtained in synapses dialyzed with control buffer (C.B., *n* = 5) and synapses exposed to Kif18A(1–453) (*n* = 4) using Mann–Whitney test. Significance level was set at *p* < 0.05. Scale bars indicate 500 nm.

Neurons contain a mixture of labile and stable microtubules, which are subject to different regulatory mechanisms^10^. If all microtubules present in the presynaptic compartment had comparable molecular properties they would be equally affected by depolymerization using SBTub3 or Kif18A(1–453) but, if they presented different degrees of stability, only the more dynamic population should be affected. A representative neuron was dialyzed with 90 nM Kif18A(1–453), causing an increase in mEPSC frequency without affecting the amplitude or shape of EPSCs and, was then prepared for serial TEM tomography. The effect of Kif18A(1–453) was characterized by an increase in mEPSC frequency (Fig. 7a). Two axosomatic and two axodendritic synapses proximal to the soma were reconstructed (Fig. 7b–e). The average volume of the identified presynaptic terminals was of 0.8 ± 0.1 μm^3^ (mean ± s.e.m.), not different from the 1.1 ± 0.3 μm^3^ found in control boutons (mean ± s.e.m., *n* = 5, *p* = 0.41, Mann–Whitney test).

Whilst microtubules found in the analyzed portions of the axon were apparently insensitive to the action of Kif18A(1–453), i.e., compare Fig. 7d, e with Fig. 3f, g, those found in the presynaptic compartment were shortened (Fig. 7f, Supplementary Data 6). The average extension of 11 ± 3 μm/terminal (*n* = 5) found in control neurons was reduced to 4 ± 1 μm/terminal (*n* = 4; *p* = 0.03, Mann–Whitney test). This result is consistent with the presence of a labile population of microtubules in the presynaptic terminal. The synchronous shortening of microtubules had an impact on the organelles present in the presynaptic compartment (Fig. 7b–e, Supplementary Movies 9–12). Although the total number and density of synaptic vesicles were not different from controls, the numbers of exo-endocytic pits and endosomes were increased by almost an order of magnitude (Fig. 7g–i). Autophagosomes were not observed in any of the control presynaptic terminals investigated but, were evident in two terminals (Fig. 7b, c) dialyzed with Kif18A(1–453), thus supporting the activation of the presynaptic endolysosomal system in conditions of intense membrane turnover^33,34^.

### Depolymerization of microtubules alters the depletion and refilling of synaptic vesicle pools

**Fig. 8:**
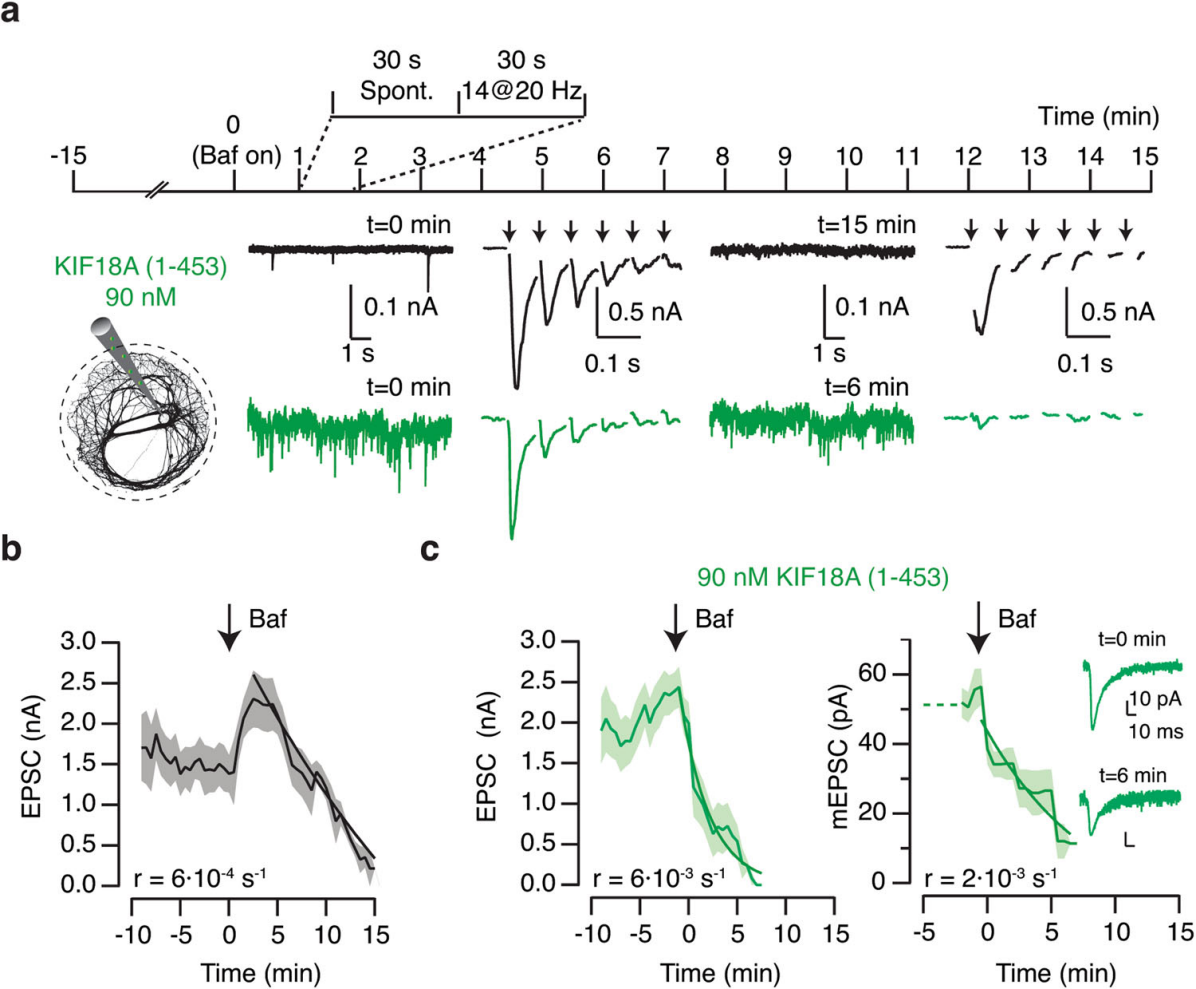
Effect of bafilomycin and microtubule depolymerization on spontaneous and evoked neurotransmitter release. **a** Neurons were dialyzed with control internal solution (black, *n* = 5) or Kif18A(1–453) (green, *n* = 6). Neurotransmission was evaluated in 1 min cycles, which consisted in a 30 s recording of spontaneous neurotransmitter release followed by a train of 14 stimuli (arrows) delivered at 20 Hz. **b** The mean amplitude of excitatory postsynaptic currents (EPSCs) decreased after application of 1 μM bafilomycin (arrow). The decay was well described by an exponential fit (black trace) that provided a rate constant (*r*). Shadowed areas illustrate s.e.m. **c** Dialysis with Kif18A(1–453) accelerated the reduction of EPSC amplitude induced by bafilomycin as well as  allowed to observe a decrease in the size of miniature EPSCs (mEPSCs). Insets show the average mEPSC obtained in the recordings of spontaneous activity displayed in (**a**).

The number of synaptic vesicles present in presynaptic terminals is regulated through local recycling mechanisms^35,36^ while also being influenced by the delivery of synaptic vesicle precursors from microtubule plus ends^6^. Vesicular proton pump inhibitors, such as bafilomycin, impede the refilling of vesicles reformed by endocytosis with neurotransmitters, thus allowing the estimation of the kinetics of the synaptic vesicle cycle. Neurons were dialyzed with Kif18A(1–453) and spontaneous and evoked neurotransmission were evaluated in cycles lasting 1 min. A 30 s recording of spontaneous neurotransmitter release was followed by the stimulation of vesicle recycling using a train of 14 stimuli delivered at 20 Hz (Fig. 8a). The possible contribution of asynchronous neurotransmitter release was eliminated by using an internal solution containing 10 mM EGTA and by excluding any neurotransmission occurred from the end of the stimulus train until the completion of the 1 min cycle.

Application of 1 μM bafilomycin induced a gradual reduction in both evoked and spontaneous neurotransmitter release (Fig. 8a). Bafilomycin was more effective suppressing EPSCs in neurons dialyzed with Kif18A(1–453) than in neurons dialyzed with control internal solution. Synaptic strength was reduced at a rate of 6 × 10^−3^ s^−1^, one order of magnitude faster than in control neurons (Fig. 8b, c, Supplementary Data 7). The high frequency of mEPSCs allowed the estimation of their amplitude, which decreased at a rate of 2 × 10^−3^ s^−1^ after bafilomycin application (Fig. 8a, c). The gradual decay observed in mEPSC amplitude suggests a leak of acetylcholine, which contrasts to results obtained for glutamatergic synaptic vesicles: they show minimal glutamate leakage in the presence of bafilomycin^37^ and are thus found in a full or empty state^38^.

The faster reduction of neurotransmitter release caused by bafilomycin in neurons dialyzed with Kif18A(1–453) argues against the possibility that mEPSCs were caused by exocytosis of synaptic vesicle precursors detached from microtubules. Precursors originate in the soma^39^ and they are therefore insensitive to the acute inhibition of the vesicular proton pump. The potential release of synaptic vesicles non-generated by local recycling mechanisms can consequently not explain an increased sensitivity to bafilomycin exposure. The enhanced effect of bafilomycin in neurons dialyzed with Kif18A(1–453) (Fig. 8b, c) is consistent with an increase in neurotransmitter release probability. The ratio between the rates of bafilomycin inhibition: r_Kif18A(1–453)_/r_cont_ describes the relationship between neurotransmitter release probabilities found in neurons dialyzed with Kif18A(1–453) and neurons dialyzed with control internal solution^38^. Consequently, microtubule depolymerization increased by ten-fold the rate of spontaneous synaptic vesicle exocytosis. Observations obtained using bafilomycin agreed with tomography data that showed a change from 3 ± 1 exo/endocytic pits (mean ± s.e.m., *n* = 5) in control neurons to 44 ± 8 exo/endocytic pits (mean ± s.e.m., *n* = 4, Fig. 7g–i) in neurons dialyzed with Kif18A(1–453).

**Fig. 9:**
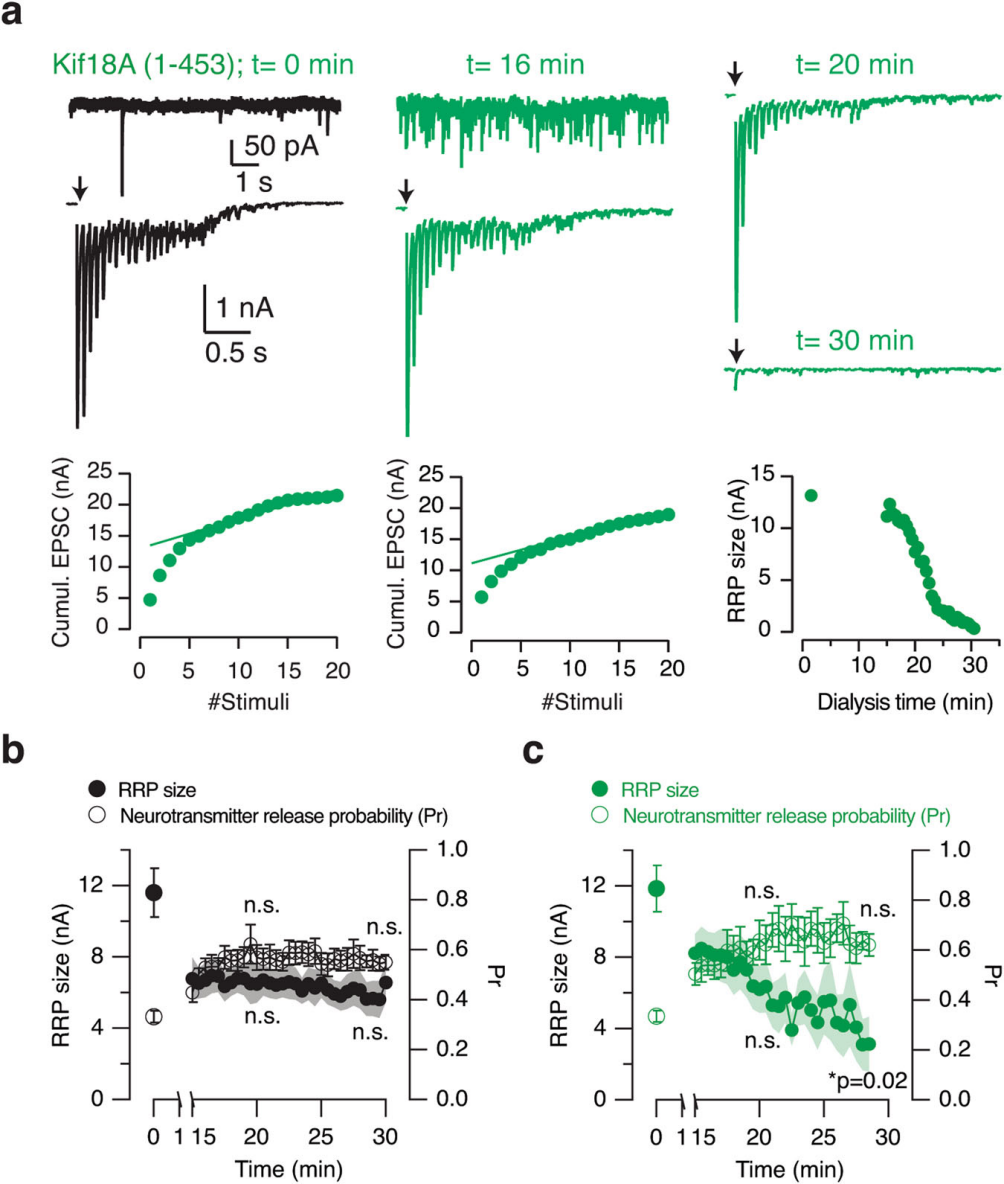
Microtubule depolymerization impairs sustained neurotransmitter release. **a** Example of the protocol applied. A neuron dialyzed with Kif18A(1–453) showed few miniature excitatory postsynaptic currents (mEPSCs) and a characteristic response to a train of 20 stimuli delivered at 20 Hz (arrow). The steady-state phase of the cumulative plot of EPSCs was fitted to a line, which allowed to calculate the size (nA) of the readily releasable pool (RRP). Upon the observation of the characteristic increase in spontaneous neurotransmitter release caused by Kif18A(1–453), which in this example occurred 16 min after establishing the whole-cell configuration, the size of the RRP was continuously evaluated in 30 s intervals. Notice the exhaustion of the RRP after the successive application of trains of stimuli. **b**, **c** Plot of the RRP size and neurotransmitter release probability (Pr, calculated as the fraction of the RRP released by the first stimulus of the train) against experimental time. The RRP size decayed in cells dialyzed with Kif18A(1–453) at a constant release probability from 15 to 30 min of experimental time. Circles and dots indicate mean. Error bars and shadowed areas indicate ±s.e.m (*n* = 8 in both groups). RRP size and Pr observed at 22 and 27 min of dialysis were compared to values obtained at 15 min of dialysis (one-way ANOVA followed by Dunnett’s multiple comparisons test). Significance level was set at *p* < 0.05.

The elevation of mEPSC frequency did not affect evoked neurotransmission triggered by seldom stimulations (Figs. 5 and 6). The absence of an effect could imply the segregation of vesicles participating in spontaneous and evoked neurotransmitter release, or a minimal depletion of synaptic vesicle pools. We thus investigated the responses to 20 Hz stimulus trains delivered in 30 s intervals to neurons dialyzed with 90 nM Kif18A(1–453). Repetitive high-frequency stimulation started 15 min after establishing the whole-cell configuration to allow for the depolymerizing activity of Kif18A (Fig. 9a, Supplementary Data 8). Dialysis either with control buffer or Kif18A(1–453) decreased the initial size of the readily releasable pool (RRP) estimated in plots of cumulative amplitude of EPSCs^40^. The RRP of control neurons reached a steady-state value that was maintained from minute 15 to 30, which contrasted to the ∼40% decrease in RRP size observed in neurons dialyzed with Kif18A(1–453) (Fig. 9a–c, Supplementary Data 8). Release probability, measured as the fraction of the RRP depleted by the first stimulus of the train, was comparable among neurons evaluated (one-way ANOVA analysis). These results illustrate a dual role of microtubules in neurotransmission as the increase in spontaneous neurotransmitter release coincided with the inability to maintain a high rate of evoked neurotransmission^7^. The depletion of recycling vesicles in the absence of action potentials could impair the refilling of the RRP if vesicles participating in spontaneous and evoked neurotransmitter release were drawn from the same pool. A reduced delivery of synaptic precursors^6^, or, an impairment of directed synaptic vesicle mobility^41^, could also contribute to the observed decrease in RRP size during high-frequency stimulation.

### Increased Stathmin-1 concentrations favor spontaneous neurotransmitter release

**Fig. 10:**
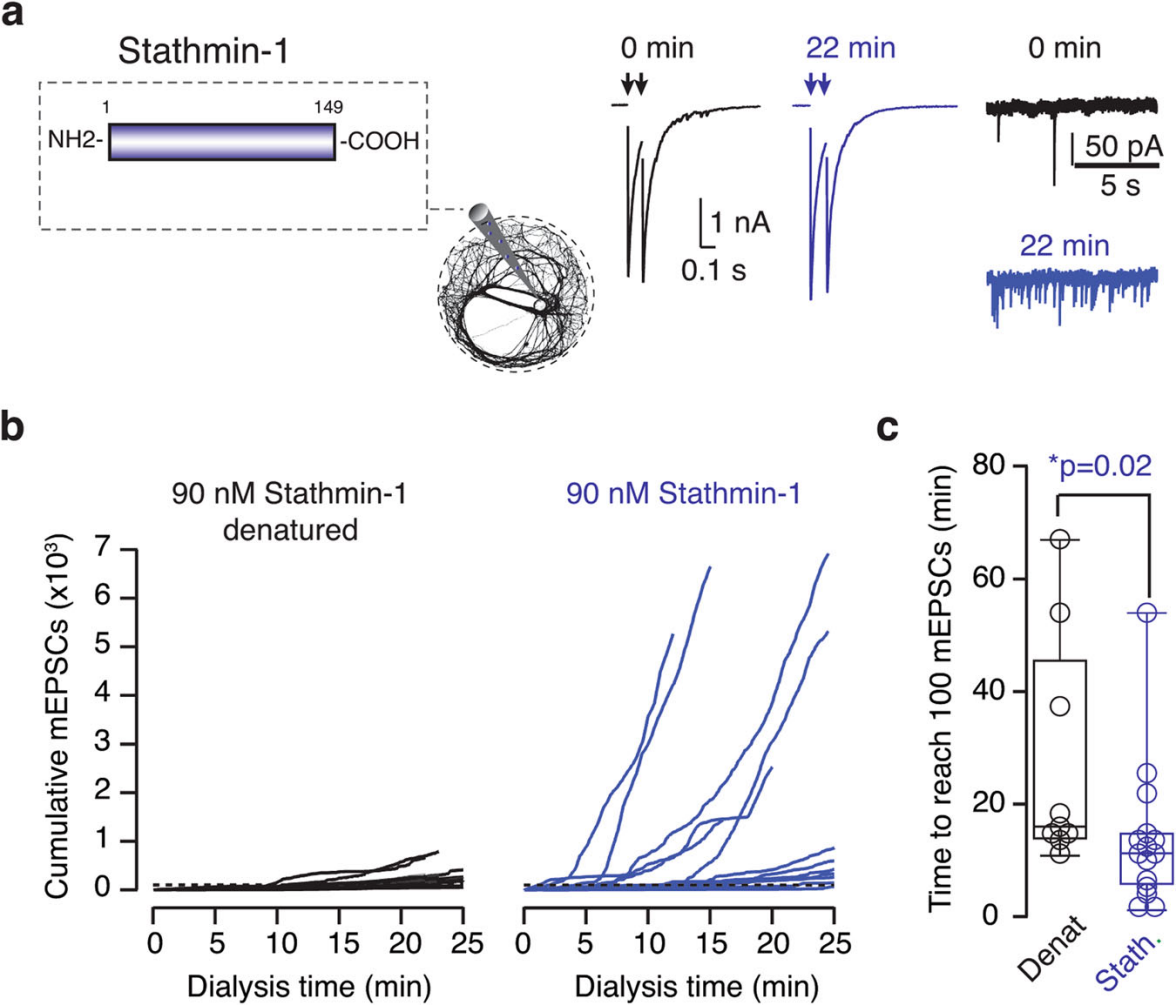
The elevation of cytosolic stathmin-1 concentration increases spontaneous neurotransmitter release. **a** Neurons were recorded in the whole-cell configuration of the patch-clamp technique when the internal solution contained 90 nM recombinant stathmin-1 (*n* = 15) or 90 nM denatured stathmin-1 (*n* = 9). Excitatory postsynaptic currents (EPSCs, arrows) and miniature EPSCs (mEPSCs) recorded in a neuron dialyzed with 90 nM stathmin-1 at the indicated times. **b** Increase in the cumulative number of mEPSCs as a function of dialysis time with stathmin-1. Each trace shows the spontaneous activity of a single neuron. Dotted lines indicate 100 mEPSCs. **c** The time required to observe 100 mEPSCs decreased in neurons dialyzed with stathmin-1. Box plot shows the median (horizontal line), 25 to 75% quartiles (boxes), and ranges (whiskers) of the experimental groups indicated. Statistical differences were evaluated using Student’s unpaired t-test. Significance level was set to *p* < 0.05.

Since Kif18A is mostly expressed in non-neuronal tissues, a key question was whether endogenous factors of the nervous system could mimic its actions on neurotransmitter release. Stathmin-1 is a cytosolic protein that promotes microtubule catastrophe, whose activity is regulated by phosphorylation and dephosphorylation^42,43^. It is widely present in the nervous system and its expression levels are maximal in the early stages of development^44^. Neurotransmission was monitored in neurons that were dialyzed with 90 nM full-length stathmin-1. Evoked responses to paired-pulse stimuli remained stable during experiment time and the frequency of mEPSCs gradually increased (Fig. 10a). Although the average effect of stathmin-1 dialysis resembled the action of Kif18A(1–453), the analysis of individual data suggested the presence of two types of responses (Fig. 10b, c, Supplementary Data 9). Half of the neurons studied showed the characteristic increase in spontaneous neurotransmitter release caused by microtubule depolymerization but, the effect was minimal and resembled controls in the other half.

The variability observed in the stathmin-1 effect on mEPSCs could be related to the actual concentration achieved in the presynaptic compartment. Both Kif18A(1–453) and stathmin-1 act at microtubule plus ends but whereas the kinesin has the capacity to move along microtubules, stathmin-1 diffuses through the cytosol. The inability of stathmin-1 to increase spontaneous neurotransmitter release in a subset of neurons could simply reflect the impossibility to reach an effective concentration in axon terminals. The action of stathmin-1 is associated to its capacity to sequester tubulin dimers and destabilize microtubule plus ends^43^, which sets the basis for regulating microtubule growth through the establishment of free tubulin concentration gradients^45^. Under this assumption, mEPSCs would increase only in those neurons more efficiently dialyzed. Alternatively, and considering the similar size of SCMs, it can be assumed that dialysis was comparable in all neurons studied. In that case, differences in the activity of stathmin-1 could be driven by inhibitory phosphorylation^43^, occurring in those neurons that did not show an elevation of mEPSC frequency. Although the data do not allow to establish a precise mechanism for stathmin-1 action, these results show that its cytosolic levels are important for spontaneous neurotransmitter release. More importantly, the effect is comparable to the action of SBTub3 and Kif18A(1–453) and therefore, reinforces the relevance of microtubule stability to presynaptic terminal function.

## Discussion

Our results reveal three fundamental aspects of the role of the microtubule network present in the presynaptic compartment: (i) microtubule plus ends are found within axon terminals and show dynamic instability, (ii) microtubule depolymerization increases spontaneous neurotransmitter release, and (iii) the integrity of microtubules is important to maintain evoked synaptic responses during high-frequency stimulation. Current findings are based in the evaluation of synaptic responses and presynaptic ultrastructure upon the induction of microtubule depolymerization in all compartments of cholinergic autaptic neurons. The results obtained agree with previous works carried out at the giant presynaptic terminal of the calyx of Held, where the microtubules contribute to the refilling of synaptic vesicle pools during high-frequency neurotransmission^7,41^. Moreover, our results illustrate that microtubule instability is important for spontaneous neurotransmitter release.

The study by automated serial electron tomography of a population of cholinergic autaptic contacts revealed the presence of many conserved features, such as size, number of active zones or synaptic vesicle density. Microtubules were present in all axon terminals investigated but their distribution was variable. Some of them extended from the axon to the presynaptic compartment while others were found exclusively within the axon terminal. This organization is consistent with the presence of a branched presynaptic network of oriented microtubules^46^ (Supplementary Fig. 1). All microtubules showed a comparable ability to tether vesicles and mitochondria but, it is possible that certain functions were segregated according to their distribution. Microtubules proximal to the axon are ideally located to carry out functions related to organelle transport and delivery of synaptic vesicle precursors^1^. In contrast, microtubules in close contact to the cytoplasmic pool of synaptic vesicles could be suited to carry out specific roles, as for example, participate in mechanisms of synaptic vesicle recycling or determine organelle mobility. Presynaptic microtubules interacted with the endosomes that were distributed in the periphery of the cytoplasmic vesicle pool. Considering the key role of the endolysosomal system in the recycling of synaptic vesicles^33,36^, it is conceivable that microtubule dynamics affects the positioning of endosomes, which could influence synaptic vesicle pools and neurotransmitter release.

The dynamism of presynaptic microtubules was supported by their variable length, which ranged from 0.2 to >1.5 μm. The probability of a sudden shortening of a given number of microtubules can be defined by a Poisson process. If presynaptic microtubules showed the rate of catastrophe of 1.14 min^−1^ found in the mitotic spindle^47^, then the probability of observing the catastrophe of a given microtubule during a one-minute period would be 0.36. The simultaneous visualization of abrupt shortenings in multiple microtubules would thus become increasingly unlikely. Assuming the stability of presynaptic microtubules is greater than those found at the bipolar spindle^10^, a synchronous catastrophe of all microtubules would be extremely rare. All presynaptic terminals investigated contained microtubules, which concurs with this theoretical scenario and, suggests that dynamic instability acts as a regulator of microtubule length rather than number. Although the continuous balance between growth and shortening would have little impact on the overall extension of the presynaptic microtubule network, the interactions with organelles would be transiently shaped by the rate of catastrophe.

The dynamism exhibited by EB3-tdTomato (Fig. 1) and the widespread presence of microtubule plus end tracking proteins in axon terminals (Fig. 2) are two evidences supporting the existence of an ongoing dynamism of presynaptic microtubules that goes beyond the structural role played in growth cones^48^. The most obvious consequence of a synchronous microtubule depolymerization induced by SBTub3 (Figs. 4 and 5), Kif18A(1–453) (Fig. 6), and stathmin-1 (Fig. 10) is the increase in spontaneous neurotransmitter release. Although current observations should be confirmed by future studies restricting  microtubule depolymerization to small axon terminals, our data confirm a relevant role of microtubules in the maintenance of synaptic vesicle pools^7,41^ and prompt to consider the interaction with microtubules as a trait of vesicles participating in the spontaneous release of neurotransmitters^49–51^.

If the stochastic occurrence of catastrophes had a direct implication on spontaneous neurotransmitter release, then the more the number of catastrophes, the more the number of miniature excitatory postsynaptic events. The maximum likelihood of catastrophes would be represented in our experimental conditions upon photoactivation of SBTub3 or dialysis of Kif18A(1–453). In both situations, a local and transient increase in soluble tubulin occurs. Vesicles clustered in the center of the presynaptic terminal could become available to interact with free tubulin dimers, for example through the ability of synaptotagmin to bind to the C-terminal region of β-tubulin^9^. Although such interaction promotes microtubule growth in vitro, and therefore could facilitate the reformation of microtubules, it is also possible to speculate that the synaptotagmin-tubulin interaction might favor neurotransmitter release through an unknown mechanism. Considering microtubule depolymerization occurred in all neuronal compartments, vesicles found in extrasynaptic locations could also contribute to the observed effect.

It is appealing to consider vesicles physically attached to microtubules as those responsible for spontaneous neurotransmitter release, however, the exponential increase in mEPSCs observed in the presence of SBTub3 or Kif18A(1–453) is inconsistent with the configuration of a defined population. The pool of synaptic vesicles supporting spontaneous neurotransmitter release is finite and represents about half the size of the resting pool^51^. Microtubules bound approximately 1–2% of cytoplasmic vesicles present in the presynaptic terminal, which is close to the size of the RRP^23,52^. A slowdown in spontaneous neurotransmitter release should thus be expected after the release of ∼200 vesicles, i.e., ∼200 mEPSCs. Current observations can be better explained if the presence of a population of labile microtubules were restricting the synaptic vesicle cycle. Tomograms revealed that polymerized microtubules participate in the positioning of endosomes and mitochondria, which could limit synaptic vesicle mobility and the kinetics of neurotransmitter release by favoring organelle crowding^53^.

Recent observations carried out in central glutamatergic synapses show the diffuse distribution of sites used for spontaneous neurotransmitter release around a central region committed to evoked neurotransmission^54^. Since a similar organization is observed in other presynaptic terminals, such as those of photoreceptors^55^, it is conceivable that axon terminals forming cholinergic autapses presented a comparable spatial relationship between sites dedicated to spontaneous and evoked neurotransmitter release. Elimination of the physical barrier established by polymerized microtubules could increase the availability of fusion competent synaptic vesicles. A putative increase in mobility and/or the arrival of vesicles mobilized from extrasynaptic locations would result in an enhanced access to release sites dedicated to spontaneous release.

Under low activity conditions, a high occupancy of the exocytic regions used for evoked neurotransmitter release can be assumed and, consequently, the enhancement of release probability associated to microtubule depolymerization could only occur by displacing secretory events to peripheral sites. In contrast, during periods of high activity, the depletion of synaptic vesicle pools is expected. Synaptic vesicle turnover is maximal and the contribution of microtubules to the replenishment of pools^7^ is impaired.

An enhancement of neurotransmitter release in the absence of action potentials could be useful in certain situations, as for example, during the assembly of neuronal circuits.  The first step in synapse formation is synaptic vesicle exocytosis^56^, which confers an important role to the elevated  spontaneous neurotransmitter release rate present during the assembly of neuronal circuits^57^. Our results prompt to consider the involvement of  microtubule instability and bring forward the contribution of stathmin-1 because it is a protein that promotes microtubule depolymerization. The levels of stathmin-1 peak during embryonic stages and decay postnatally^58^, which could set a molecular link to the high rate of spontaneous neurotransmitter release observed during the development of the nervous system.

## Methods

### Cell culture and molecular biology

Experimental procedures were approved by the Department of Environment of the Generalitat de Catalunya. SCMs from superior cervical ganglion neurons were prepared using postnatal day 0 (P0) to P2 Sprague Dawley rats^15^. Briefly, medium containing all dissociated ganglionic cells was placed in a 100-mm-diameter culture dish for 60 min at 37 °C. At the end of this preplating period, ≥95% of non-neuronal cells were found to be adhered to the dish, but most neurons remained in suspension. Medium was then collected, and neurons seeded at 2500 cells·mL^−1^ on 15 mm coverslips containing 10–20 collagen microdrops of 100–400 μm diameter. Culture medium was DMEM/F12 [1:1] containing 2.5% fetal bovine serum, 2.5% rat serum (prepared in the animal care facility of the Campus of Bellvitge, University of Barcelona), 5 nM NGF (Alomone Labs, Jerusalem, Israel, N-100), 2 nM CNTF (Alomone Labs, C-245), and 25 U/ml penicillin/streptomycin at 37 °C and 8% CO_2_.

EB3-tdTomato was a gift from Erik Dent (Addgene plasmid # 50708; http://n2t.net/addgene:50708; RRID:Addgene_50708)^18^. The coding sequence of EB3-tdTomato was cloned into pWPXL (Addgene plasmid # 12257; http://n2t.net/addgene:12257; RRID:Addgene_12257)) by replacing EGFP using MluI-HF and NdeI. For lentivirus production, HEK 293T cells were transfected by the calcium phosphate method following methods described by Didier Trono (http://tronolab.epfl.ch/lentivectors) with pMD2G (Addgene plasmid # 12259; http://n2t.net/addgene:12259; RRID:Addgene_12259), pCMVR8.74 (Addgene plasmid # 22036; http://n2t.net/addgene:22036; RRID:Addgene_22036), and pWPXL. Two days later, culture medium containing lentiviral particles was collected in 3 rounds at 8 h intervals, kept at 4 °C, and centrifuged at 500 × *g*. Supernatants were distributed in aliquots and stored at −80 °C.

### Electrophysiology

All experiments were performed in the whole-cell configuration of the patch-clamp mode using neurons microcultured for 18–22 DIV. Typical resistances of pipettes used for recordings were 3–5 MΩ when filled with standard internal solution composed of the following (in mM): 130 K-gluconate, 4 MgCl_2_, 1 EGTA, 10 HEPES, 3 Na_2_ATP, 1 NaGTP, pH 7.2, 290 mOsm/kg. External solution contained (in mM): 130 NaCl, 5 KCl, 2 MgCl_2_, 10 HEPES-hemisodium salt, and 10 glucose, pH 7.4. The final 2 mM CaCl_2_ concentration was always achieved by dilution from a 1 M stock. All salts were from Sigma-Aldrich (St. Louis, MO). Before the addition of glucose and CaCl_2_, the osmolality of the external solution was adjusted to 290 mOsm/kg. Experiments were performed at room temperature (23 °C).

Recordings were made using an Axopatch-1D patch-clamp amplifier (Molecular Devices) under the control of an ITC-18 board (Instrutech Corp) driven by mafPC (courtesy of M. A. Xu-Friedman, University at Buffalo, NY). Neurons were clamped at −60 mV and stimulated by a 1–2 ms depolarization step that drove membrane potential to 0 mV. The presence of functional autaptic synapses was identified by the generation of excitatory postsynaptic currents (EPSCs). Further details of neurotransmission in SCMs are described elsewhere^17,23,59,60^. Experimental time typically ranged from 20 to 50 min. Recordings were finished if holding current values were larger than 0.5 nA.

Recombinant Kinesin Family, Member 18A (1–453) (KIF18A) (Cloud-Clone Corp., RPF518Hu01) was reconstituted in Tris 20 mM, NaCl 150 mM to a concentration of 1 mg/ml and diluted in standard intracellular solution for electrophysiological recordings. For control conditions, the reconstitution buffer was added instead (1:40, same dilution as for 90 nM Kif18A). His tagged human Stathmin-1 prepared in a buffered aqueous glycerol solution (SRP5144, Sigma-Aldrich, St. Louis, MO) was diluted in standard internal solution at a final concentration of 90 nM. Stathmin-1 thermally denaturalized for 30 min at 100 °C was used as control.

### Photoactivation of SBTub3

SCMs were incubated in recording external solution with 0.15% DMSO cosolvent, with or without 15 μM SBTub3, for 12 min in the dark followed by 2 min of extensive wash. *E* → *Z* photoactivation was performed in an inverted Olympus IX-50 microscope using a Hg arc lamp (100 W) as light source through an air 60x objective (LCPlanFI 60x/0.7, Olympus). Neurons were exposed to two 410 nm light pulses of 3 s delivered at a 30 s interval (excitation filter ET410/40, Chroma Technology Corp., VT). Experiments were always performed under green transmission light (excitation filter ET545/25, Chroma Technology Corp., VT) to prevent photoactivation during basal conditions.

### Immunocytochemistry and STED microscopy

For visualization by STED microscopy high precision glass coverslips (#1.5, Marienfeld, 0117550) containing SCMs were fixed for 15 min in 2% PFA prepared in 1x PBS. They were incubated overnight at 4 °C with a monoclonal anti-EB3 antibody (1:100, Invitrogen, MA1-72525) combined with the polyclonal anti-synapsin I primary antibody (1:250, Merck Millipore, AB1543P). Appropriate secondary antibodies labeled with Alexa Fluor Plus-594 (1:100) and Atto-647N (1:100) were used for fluorescent staining of EB3 and synapsin I, respectively. Coverslips were mounted with ProLong Gold antifade reagent (Thermo Fisher Scientific).

Tau-STED 3D datasets were acquired with a Leica TCS SP8 STED 3X FALCON (Leica Microsystems, Mannheim, Germany) on a DMI8 stand using a 100×/1.4 NA HC CS2 PL APO objective. A pulsed supercontinuum light source (white laser) with a repetition rate of 80 MHz and with the FLIM acquisition module enabled Alexa Fluor Plus-594 and Atto-647N excitation. The white laser was set at 587 nm and 633 nm, respectively. A 775 nm pulsed depletion (STED) laser was added with no pulse delay at 20% intensity. Detection was performed with hybrid detectors (HyDs) set between 595 and 637 nm for Alexa Fluor Plus-594 (EB3) and between 642 and 750 nm for Atto-647N (synapsin I). Scanner speed was set bidirectionally at 600 Hz and images were taken with 4x line repetitions. Zoom was set at 4 and pixel size was defined according to the depletion power to 22 nm, resulting in 1304 × 1304 pixel images. The channels of 3D stacks were taken sequentially in a stack-by-stack acquisition mode and with a z-step of 156 nm. A total of 14 to 27 z slices were obtained depending on the volume of the structure of interest. After acquisition, the lifetime-containing datasets were processed with the Tau-STED module, which exploits the fluorescence lifetime gradient induced by the STED beam to separate the STED signal from background. Depending on the amount of the remaining background, a post-acquisition time gating was applied to remove it.

### Correlative electrophysiology and electron tomography

For correlative electrophysiology and electron tomography experiments, neurons grown in thermanox coverslips (Thermo Scientific, 174969) were recorded, micrographed, and fixed in 1.8% glutaraldehyde prepared in 0.1 M sodium cacodylate buffer for 20 min at RT and 20 min at 4 °C. SCMs were postfixed with freshly made 1% osmium tetroxide/1.5% potassium ferricyanide, dehydrated in alcohol series and embedded in EPON resin.

Sections of 250 nm thickness were placed on Formvar-coated slot grids and 10 nm PAG gold fiducials were added to the grids to be used later for alignment and reconstruction of the tomograms. Grids were post-stained and imaged on a Tecnai F30 (Thermo Fisher Scientific, Waltham, MA, USA) transmission electron microscope equipped with a Gatan OneView camera (Gatan, Inc., Pleasanton, USA). Precise target positions were manually selected using the SerialEM software^61^ and acquired by dual-axis tomography (−60° to +60° per axis; increment of 1°) at 12,000x magnification (2.05 nm/px). The tilt series were reconstructed on a high-performance compute cluster using IMOD’s automated batch reconstruction^62,63^ and joined using etomo to yield the final volumes. Acquired images were rescaled by a two-fold binning factor to yield a pixel size of 1.303 nm. Semiautomated segmentation for 3D modeling, 3D visualization, and quantitative measurements were performed with IMOD software. Tomogram images were subjected to noise reduction by using the Gaussian filter.

### Imaging of EB3-tdTomato comets

Neurons were infected with lentiviruses to express EB3-tdTomato. Fluorescence was obvious 36-48 h after infection. Coverslips were mounted on an RC-25 imaging chamber (Warner Instruments) and observed in a Zeiss LSM 880 confocal microscope (Carl Zeiss AG, Oberkochen, Germany). A confocal plane was imaged using a 63X oil immersion objective LD LCI Plan-Apochromat (1.2 N.A.). TdTomato was excited at 555 nm and fluorescence was collected using a GaAsp detector. The visualization of EB3 comets was carried out in 1024 × 1024 pixel images obtained at a resolution of 22.7 pixels·μm^−1^ (voxel size 0.044 × 0.044 × 0.499 μm^3^). 2D confocal sections were collected at 0.8 Hz for 2 min. Movies were exported to Image J and Fiji. Photobleaching was corrected before doing the analysis of observed trajectories.

### Statistics and reproducibility

Statistical analysis was carried out using parametric or non-parametric tests after evaluating experimental groups with the Kolmogorov-Smirnov test. Differences were established by one-way ANOVA followed by Dunnett’s multiple comparisons test. Differences between two groups were established by the unpaired Student’s t-test or Mann–Whitney test. *P* values <0.05 were considered as significant. All experiments investigating synaptic responses used data from six or more individual SCMs, which were established in three or more different culture procedures.

### Reporting summary

Further information on research design is available in the Nature Portfolio Reporting Summary linked to this article.

## Supplementary information

Llobet_Peer Review File

Supplementary Information

Description of Additional Supplementary Files

Supplementary Data 1

Supplementary Data 2

Supplementary Data 3

Supplementary Data 4

Supplementary Data 5

Supplementary Data 6

Supplementary Data 7

Supplementary Data 8

Supplementary Data 9

Supplementary Movie 1

Supplementary Movie 2

Supplementary Movie 3

Supplementary Movie 4

Supplementary Movie 5

Supplementary Movie 6

Supplementary Movie 7

Supplementary Movie 8

Supplementary Movie 9

Supplementary Movie 10

Supplementary Movie 11

Supplementary Movie 12

Reporting Summary

## Data Availability

Electron tomography data and segmentations are available at the Electron Microscopy Public Image Archive (EMPIAR), identifier EMPIAR-11445. Numerical source data used for generating the graphs presented in this study are available as Supplementary Data 1–9. The rest of data supporting the findings of this study are available from the corresponding author upon reasonable request.
