# Supplementary material for: Microtubule depolymerization contributes to spontaneous neurotransmitter release in vitro
Source: Commun Biol. 2023 May 5;6:488. doi: 10.1038/s42003-023-04779-1 (PMC10163034; doi:10.1038/s42003-023-04779-1)
Supplement: Supplementary file 2 — Supplementary Information [file 42003_2023_4779_MOESM2_ESM.pdf]

Supplementary Information

Microtubule depolymerization contributes to spontaneous neurotransmitter release in vitro

Cecilia D. Velasco[1,2], Rachel Santarella-Mellwig[3], Martin Schorb[3], Li Gao[4], Oliver Thorn-Seshold[4] and Artur Llobet[1,2,*]

[1] Laboratory of Neurobiology, Department of Pathology and Experimental Therapy, Institute of Neurosciences, University of Barcelona, 08907 L'Hospitalet de Llobregat, Barcelona, Spain

[2] Bellvitge Biomedical Research Institute (IDIBELL), 08907 L'Hospitalet de Llobregat, Barcelona, Spain

[3] Electron Microscopy Core Facility, European Molecular Biology Laboratory (EMBL), Meyerhofstrasse 1, 69117 Heidelberg, Germany

[4] Department of Pharmacy, Ludwig-Maximilians University of Munich; Munich 81377, Germany

* Corresponding Author:
Artur Llobet
Laboratory of Neurobiology
Department of Pathology and Experimental Therapy
Faculty of Medicine and Health Sciences, University of Barcelona
08907 L'Hospitalet de Llobregat
Barcelona, Spain
E-mail: allobet@ub.edu
Phone: +34-934024279
ORCID: 0000-0001-5797-6782

Supplementary Figure 1

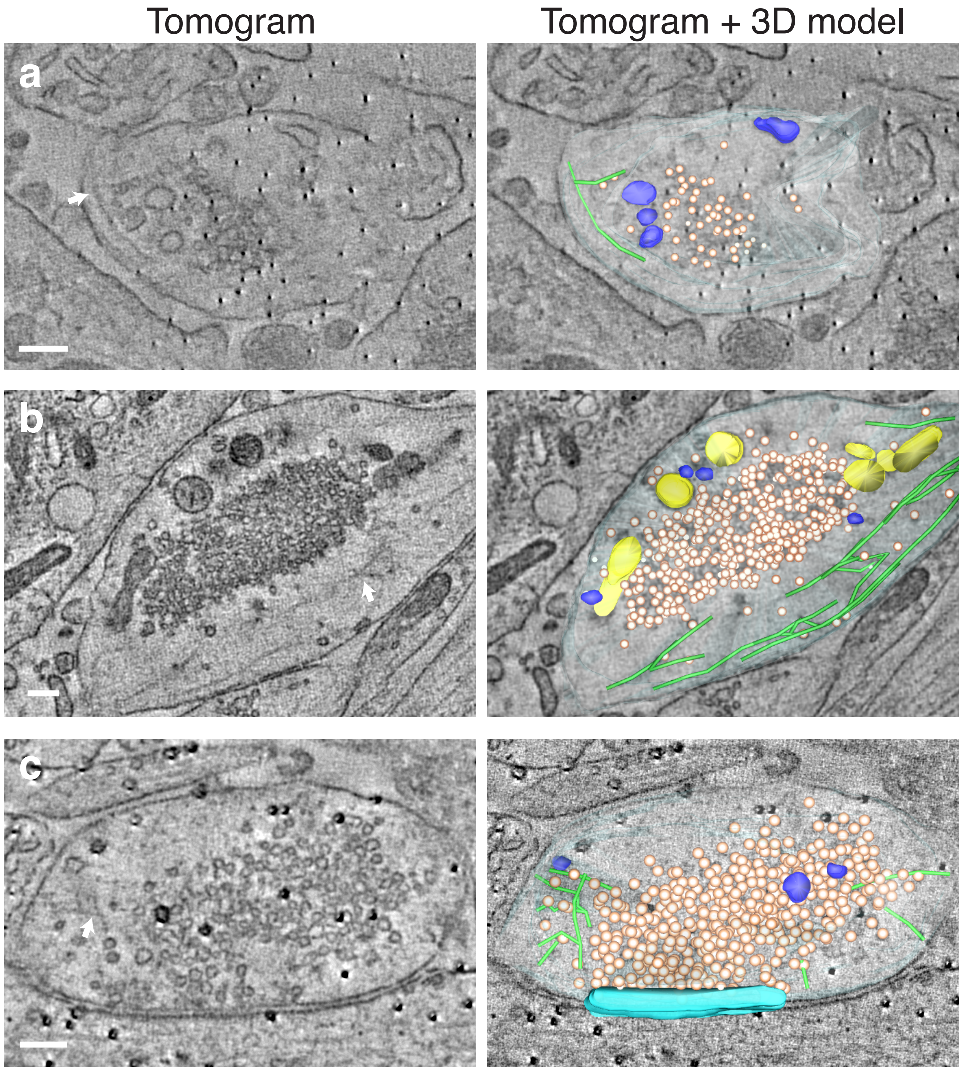

Examples of microtubules branching within the presynaptic compartment. Left, images of three different presynaptic terminals obtained by electron tomography. Arrows indicate the position of microtubules that show branching. Right, 3D model associated to the images shown indicating the presence of mitochondria (yellow), endosomes (blue), active zones (cyan) and microtubules (green). Scale bars indicate 200 nm

Supplementary Figure 2

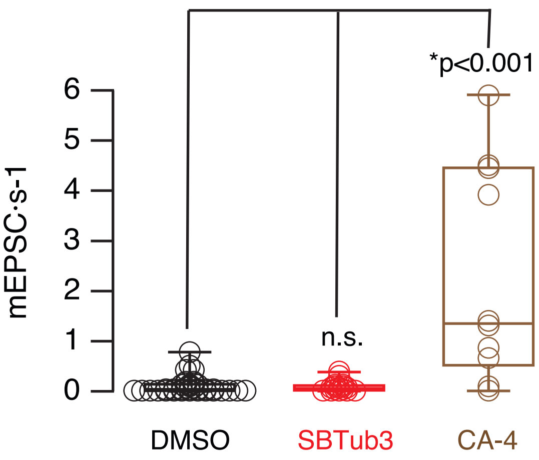

Effect of combretastatin A-4 on spontaneous neurotransmission. Single Cell Microcultures (SCMs) were incubated for 12 minutes with 0.15% DMSO (n=36), 15 µM *E*-SBTub3 (in the dark, n=16) and 15 µM combretastatin A-4 (CA-4, n=10) and abundantly washed for 2 min. The frequency of spontaneous neurotransmitter release was evaluated during 3 minutes. SCMs incubated with combretastatin A-4 showed a more than ten-fold increase in the frequency of mEPSCs while neurons incubated with *E*-SBTub3 (inactive) did not. Box plot shows the median (horizontal line), 25 to 75% quartiles (boxes), and ranges (whiskers) of the experimental groups indicated. Comparisons were established by one way ANOVA followed by Dunnett's multiple comparison test.

Supplementary Figure 3

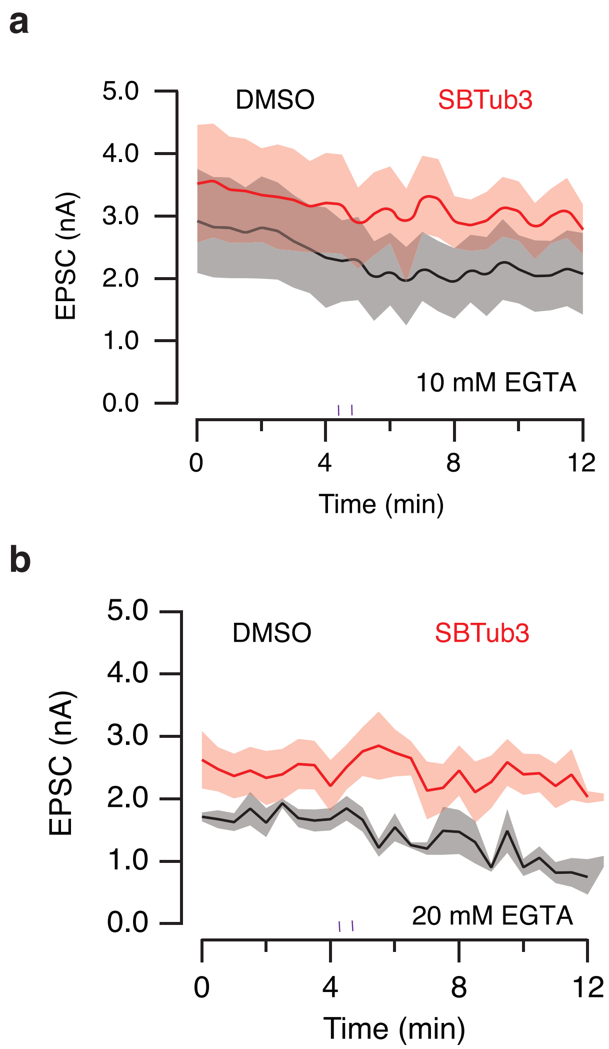

Photoactivation of SBTub3 (*E*→*Z*) did not affect evoked neurotransmission in the presence of a strong calcium buffer. a,b) Mean synaptic strength was not modified by *E*-SBTub3 photoactivation to *Z*-SBTub3 when neurons were dialyzed with internal solution containing 10 mM or 20 mM EGTA. Shadowed areas indicate s.e.m. Experimental conditions: neurons incubated with DMSO: 10 mM EGTA (n=4), 20 mM EGTA (n=10) and 15 µM *E*-SBTub3: 10 mM EGTA (n=4), 20 mM EGTA (n=7). Photoactivation was carried out according to standard protocol as in Figs 4 and 5.

Supplementary movies

- **Supplementary Movie 1.** Live imaging of EB3-tdTomato in a single cell cholinergic microculture. The imaged region corresponds to the periphery of the cell body. Images were acquired at a frame rate of 0.8 Hz. White arrows indicate axonal EB3 comets. Green arrows show the transient appearance of EB3 comets in perisomatic locations. The total time imaged is 126 s. Details on the movie can be found in text and legend associated to Figs.1b-g.
- **Supplementary Movie 2.** Tomogram of the presynaptic terminal establishing an axosomatic synapse shown in Fig. 3b.
- **Supplementary Movie 3.** 3D model and the corresponding tomogram of the presynaptic terminal shown in Fig. 3b. Synaptic vesicles (orange), microtubules (green), endosomes (blue), mitochondria (yellow) and active zone (cyan).
- **Supplementary Movie 4.** 3D reconstruction of the presynaptic terminal establishing an axodendritic synapse shown in Fig. 3c. Synaptic vesicles (orange), microtubules (green), endosomes (blue), mitochondria (yellow) and active zones (cyan).
- **Supplementary Movie 5.** 3D reconstruction of the presynaptic terminal establishing an axodendritic synapse shown in Fig. 3d. Synaptic vesicles (orange), microtubules (green), endosomes (blue), mitochondria (yellow) and active zones (cyan).
- **Supplementary Movie 6.** 3D reconstruction of the presynaptic terminal establishing an axodendritic synapse shown in Fig. 3e. Synaptic vesicles (orange), microtubules (green), endosomes (blue), mitochondria (yellow) and active zones (cyan).
- **Supplementary Movie 7.** 3D reconstruction of the presynaptic terminal establishing an axosomatic synapse shown in Fig. 3f. Synaptic vesicles (orange), microtubules (green), endosomes (blue), mitochondria (yellow) and active zones (cyan).
- **Supplementary Movie 8.** 3D reconstruction of the presynaptic terminal establishing an axosomatic synapse shown in Fig. 3g. Synaptic vesicles (orange), microtubules (green), endosomes (blue), mitochondria (yellow) and active zones (cyan).
- **Supplementary Movie 9.** 3D reconstruction of the presynaptic terminal establishing an axosomatic synapse shown in Fig. 7b. The neuron was dialyzed with Kif18A(1-453). Synaptic vesicles (orange), microtubules (light green), endosomes (blue), autophagosomes (dark green), mitochondria (yellow) and active zones (cyan).
- **Supplementary Movie 10.** 3D reconstruction of the presynaptic terminal establishing an axosomatic synapse shown in Fig. 7c. The neuron was dialyzed with Kif18A(1-453). Synaptic vesicles (orange), microtubules (light green), endosomes (blue), autophagosomes (dark green), mitochondria (yellow) and active zones (cyan).
- **Supplementary Movie 11.** 3D reconstruction of the presynaptic terminal establishing an axodendritic synapse shown in Fig. 7d. The neuron was dialyzed with Kif18A(1-453). Synaptic vesicles (orange), microtubules (light green), endosomes (blue), mitochondria (yellow) and active zones (cyan).
- **Supplementary Movie 12.** 3D reconstruction of the presynaptic terminal establishing an axodendritic synapse shown in Fig. 7e. The neuron was dialyzed with Kif18A(1-453). Synaptic vesicles (orange), microtubules (light green), endosomes (blue), mitochondria (yellow) and active zones (cyan).
