# Supplementary material for: Microtubule depolymerization contributes to spontaneous neurotransmitter release in vitro
Source: Commun Biol. 2023 May 5;6:488. doi: 10.1038/s42003-023-04779-1 (PMC10163034; doi:10.1038/s42003-023-04779-1)
Supplement: Supplementary file 3 — Description of Additional Supplementary Files [file 42003_2023_4779_MOESM3_ESM.pdf]

Description of Additional Supplementary Files

File name: Supplementary Data 1

Description: source data for Fig. 2d

File name: Supplementary Data 2

Description: source data for Fig. 3h

File name: Supplementary Data 3

Description: source data for Figs. 4b and 4c

File name: Supplementary Data 4

Description: Qu source data for Fig.5a, 5c, 5d, 5e and 5f

File name: Supplementary Data 5

Description: source data for Fig.6b and 6c

File name: Supplementary Data 6

Description: source data for Fig.7f and 7i

File name: Supplementary Data 7

Description: source data for Fig.8b and 8c

File name: Supplementary Data 8

Description: source data for Fig.9a, Fig. 9b and Fig.9c

File name: Supplementary Data 9

Description: source data for Fig.10b and 10c

File name: Supplementary Movie 1

Description: Live imaging of EB3-tdTomato in a single cell cholinergic microculture. The imaged region corresponds to the periphery of the cell body. Images were acquired at a frame rate of 0.8 Hz. White arrows indicate axonal EB3 comets. Green arrows show the transient appearance of EB3 comets in perisomatic locations. The total time imaged is 126 s. Details on the movie can be found in text and legend associated to Figs.1b-g.

File name: Supplementary Movie 2

Description: Tomogram of the presynaptic terminal establishing an axosomatic synapse shown in Fig. 3b.

File name: Supplementary Movie 3

Description: 3D model and the corresponding tomogram of the presynaptic terminal shown in Fig. 3b. Synaptic vesicles (orange), microtubules (green), endosomes (blue), mitochondria (yellow) and active zone (cyan).

File name: Supplementary Movie 4

Description: 3D reconstruction of the presynaptic terminal establishing an axodendritic synapse shown in Fig. 3c. Synaptic vesicles (orange), microtubules (green), endosomes (blue), mitochondria (yellow) and active zones (cyan).

File name: Supplementary Movie 5

Description: 3D reconstruction of the presynaptic terminal establishing an axodendritic synapse shown in Fig. 3d. Synaptic vesicles (orange), microtubules (green), endosomes (blue), mitochondria (yellow) and active zones (cyan).

File name: Supplementary Movie 6

Description: 3D reconstruction of the presynaptic terminal establishing an axodendritic synapse shown in Fig. 3e. Synaptic vesicles (orange), microtubules (green), endosomes (blue), mitochondria (yellow) and active zones (cyan).

File name: Supplementary Movie 7

Description: 3D reconstruction of the presynaptic terminal establishing an axosomatic synapse shown in Fig. 3f. Synaptic vesicles (orange), microtubules (green), endosomes (blue), mitochondria (yellow) and active zones (cyan).

File name: Supplementary Movie 8

Description: 3D reconstruction of the presynaptic terminal establishing an axosomatic synapse shown in Fig. 3g. Synaptic vesicles (orange), microtubules (green), endosomes (blue), mitochondria (yellow) and active zones (cyan).

File name: Supplementary Movie 9

Description: 3D reconstruction of the presynaptic terminal establishing an axosomatic synapse shown in Fig. 7b. The neuron was dialyzed with Kif18A(1-453). Synaptic vesicles (orange), microtubules (light green), endosomes (blue), autophagosomes (dark green), mitochondria (yellow) and active zones (cyan).

File name: Supplementary Movie 10

Description: 3D reconstruction of the presynaptic terminal establishing an axosomatic synapse shown in Fig. 7c. The neuron was dialyzed with Kif18A(1-453). Synaptic vesicles (orange), microtubules (light green), endosomes (blue), autophagosomes (dark green), mitochondria (yellow) and active zones (cyan).

File name: Supplementary Movie 11

Description: 3D reconstruction of the presynaptic terminal establishing an axodendritic synapse shown in Fig. 7d. The neuron was dialyzed with Kif18A(1-453). Synaptic vesicles (orange), microtubules (light green), endosomes (blue), mitochondria (yellow) and active zones (cyan).

File name: Supplementary Movie 12

Description: 3D reconstruction of the presynaptic terminal establishing an axodendritic synapse shown in Fig. 7e. The neuron was dialyzed with Kif18A(1-453). Synaptic vesicles (orange), microtubules (light green), endosomes (blue), mitochondria (yellow) and active zones (cyan).
