# Supplementary material for: Microtubule depolymerization contributes to spontaneous neurotransmitter release in vitro
Source: Commun Biol. 2023 May 5;6:488. doi: 10.1038/s42003-023-04779-1 (PMC10163034; doi:10.1038/s42003-023-04779-1)
Supplement: Supplementary file 1 — Llobet_Peer Review File [file 42003_2023_4779_MOESM1_ESM.pdf]

nature portfolio

Peer Review File

Microtubule depolymerization contributes to spontaneous neurotransmitter release in vitro

Open Access This file is licensed under a Creative Commons Attribution 4.0 International License, which permits use, sharing, adaptation, distribution and reproduction in any medium or format, as long as you give appropriate credit to the original author(s) and the source, provide a link to the Creative Commons license, and indicate if changes were made. In the cases where the authors are anonymous, such as is the case for the reports of anonymous peer reviewers, author attribution should be to 'Anonymous Referee' followed by a clear attribution to the source work. The images or other third party material in this file are included in the article's Creative Commons license, unless indicated otherwise in a credit line to the material. If material is not included in the article's Creative Commons license and your intended use is not permitted by statutory regulation or exceeds the permitted use, you will need to obtain permission directly from the copyright holder. To view a copy of this license, visit http://creativecommons.org/licenses/by/4.0/.

Reviewers' comments:

Reviewer #1 (Remarks to the Author):

The authors analyze here the influence of microtubule-perturbing reagents on synaptic release. Microtubules have long been seen as simply routes of vesicle transport, with little influence on synaptic release. The authors find here that their manipulation induces a significant increase in spontaneous release, which is a very interesting result.
I only have a few questions:
a) The authors suggest that the application of soluble tubulin to the synapses depolymerizes the existing microtubules (Fig. 2, 3). Has the depolymerization been demonstrated? Why would it take place?
b) In principle, the simplest interpretation of the results presented here is that soluble tubulin, either coming from depolymerized microtubules or from exogenously added tubulin, enhances synaptic release. The authors should comment on this in their discussion.
c) Figure 8 is confusing. Why did the authors combine stimulation and periods of spontaneous release recordings? As the KIF18A treatment induces an increase in spontaneous release, it should result in a relatively rapid loss of EPSC amplitude also in the absence of stimulation, assuming that the same vesicles participate in spontaneous and in stimulation-evoked release. Could the authors proof this situation, by repeating the experiments from Fig. 8 in the absence of 14-AP/20Hz trains, but rather giving only single APs at rare intervals?

Reviewer #2 (Remarks to the Author):

Review of Valesco et al.

This is an interesting manuscript focused on how microtubules in presynaptic endings affect synaptic vesicle release in autaptic synapses of superior cervical ganglion single-cell microcultures. However, even though the authors conduct solid electrophysiology there is little mechanistic insight gained and many parts of the manuscript are overinterpreted. A strength of the paper is that they use relatively newly developed mechanism to depolymerize microtubules with the photochemical SBTub3, that can rapidly depolymerize microtubules upon exposure of blue light, in conjunction with whole cell recording of EPSCs/mEPSCs. They combine these experiments with fixed cell STED imaging, EM tomography and dialysis of tubulin and a depolymerizing motor protein in the recording pipet, which allows them to clearly probe the association of microtubules and synaptic vesicle release. However, it is unclear if microtubule polymerization/depolymerization and spontaneous neurotransmitter release are directly or indirectly linked. For example, does depolymerization of microtubules result in the release of microtubule binding factors that influence spontaneous release? The authors suggest that depolymerization of microtubules do not release synaptic vesicles directly, that then fuse spontaneously, which is in line with their experimental results. However, in the discussion they suggest that microtubules may act as a brake on the synaptic vesicle cycle – how would this be accomplished?

Overall, this is an intriguing manuscript, but their overinterpretation of many of their results and their lack of mechanistic insight into how microtubules affect spontaneous release diminish the impact of their results. Specific comments are below.

-It would be useful to mention that these are superior cervical ganglion cultures at the beginning of the Results section and indicate that this type of neuron actually makes autaptic synapses in vivo, if that is the case. If these neurons do not normally form autaptic synapses, how is this work relevant to the normal functioning of neurons?

-The authors never show any live-cell imaging with EB3. The antibody labeling of EB3 and synapsin conducted with STED microscopy in Fig. 1 is useful but does not give a sense of dynamics of microtubules in presynaptic structures. Live cell imaging of EB3 during their manipulations would provide important information regarding the effect of their manipulations (tubulin/Kif18A dialysis and SBTub3 activation).

-The dialysis of tubulin into neurons in Fig. 2 and 3 is intriguing and the negative result with denatured tubulin is a good control. However, there are several problems with this experiment. 1) Do the authors have any proof that this technique indeed promotes microtubule polymerization, as they indicate? Live imaging with EB3 would need to be done to back up their assumption. 2) It is odd that this technique affects EPSCs but has little effect on mEPSCs. This is opposite to what is found by microtubule depolymerization with SBTub3 and dialysis with Kif18A later in the paper – these experiments only affect mEPSCs, but not EPSCs. 3) They propose that the frequency of spontaneous neurotransmitter release is unaltered, but only show two traces (Fig. 3d). Whereas they show the population quantification of mEPSC amplitudes in Fig. 3e. 4) In lines 142-146 they indicate that "…the presynaptic compartment remained largely inaccessible to newly polymerized tubulin…." How can they assume this? They subsequently indicate "…neurons can rapidly potentiate neurotransmission by synthesizing new tubulin monomers…." This is just conjecture/overinterpretation of their results.

-In line 215 they write that "Kif18A is not present in the cytosol of SCMs…" but do not show any data or refer to any studies showing this. Then in line 235 they write that they "demonstrate that acute depolymerization of microtubule plus ends by endogenous factors…". Do they mean exogenous factors? Presumably, KIF18A and SBTub3 are not endogenous.

-In line 239 they title the section "Microtubules dynamically interact with synaptic vesicles". This is an overinterpretation. They only show EM-tomography reconstructions. They may conclude that microtubules associate with synaptic vesicles, but dynamic interaction cannot be assumed.

-In line 183 they write that inactive E-SBTub3 is used. Shouldn't this be active E-SBTub3?

-There are several grammatical errors throughout the manuscript. It would benefit from the careful inspection by someone whose primary language is English.

Reviewer #3 (Remarks to the Author):

Using cholinergic autaptic culture preparation from rodent SCG, Velasco et al tested the effect of microtubule-depolymerizing reagents, whole-cell applied into cell bodies on evoked and spontaneous miniature EPSCs, and found them increase mEPSC frequency. Since one of the MT-depolymerizers accelerated the rundown of evoked EPSC amplitude in the presence of bafilomycin, authors conclude that MT depolymerization increases vesicle cycling rate.
This is a challenging study asking an interesting question as to the regulatory role of MTs in presynaptic terminals. Unfortunately, however, little reliable direct evidence is provided to support the authors' conclusion. Main problems are as follows:

(1) Kif18A has no effect on the evoked EPSC amplitude, but unlike what is written in the text (189-191), SBTub3 apparently enlarges EPSC and decreases the paired-pulse ratio (Fig 5c,d) suggestive of increased release probability, although no statistical evaluation is made for the difference from controls.
(2) In bafilomycin experiments, mEPSC amplitude is shown to decrease by ~80% (Fig 8c), which is inconsistent with Ikeda & Bekkers (2009) reporting that mEPSC size is unchanged in bafilomycin although it is wrongly quoted in the text (307).
(3) Acceleration of the EPSC rundown in bafilomycin (Fig 8c) can arise from a reduction in the number of recycling vesicles even without "acceleration in the cycling rate" (37-38).
(4) Surprisingly, no data is provided as to the number of synapses formed within a cell. Since mEPSCs are recorded from cell body, their frequency should reflect summed events from individual boutons.
(5) Dynamic changes of MTs (30, 39,74, 338, 339) in the terminal are not shown. Real time, or at least time-lapse imaging, is necessary.
(6) "Synchronous" MT depolymerization by SBTub3 (32) is not shown in the bouton.
(7) Postsynaptic effects of tubulin (Figs 2 and 3) seem unrelated to the main results.
(8) It is unclear whether the photo-switchable MT depolymerizer SBTub3 is giving any merit to this study.
(9) Kif18A (1-453) is utilized as a MT-depolymerizing tool but does not "highlight the relevance of physiological mechanism" (236) particularly since it is normally unexpressed in the bouton (214, 215).
(10) It is unclear whether the enhancement of mEPSC frequency upon MT depolymerization can play any physiological role.

Reviewer #1 (Remarks to the Author):
We thank the reviewer for their time and careful consideration of our work.

The authors analyze here the influence of microtubule-perturbing reagents on synaptic release. Microtubules have long been seen as simply routes of vesicle transport, with little influence on synaptic release. The authors find here that their manipulation induces a significant increase in spontaneous release, which is a very interesting result.

Our original paper evaluated how neurotransmission was affected by an acute increase in the number of microtubules as well as by the synchronous depolymerization of microtubules. We found that addressing all reviewers' comments would significantly increase the length of the manuscript, which already contained 8 figures. Since most of the criticisms were related to microtubule depolymerization, we decided to exclusively focus on this aspect to elaborate a clearer, more specific work. This new version of the paper contains 10 figures. We have done the following four new sets of experiments: 1) live imaging of EB3-tdTomato comets (Fig. 1); 2) automated serialEM of a neuron dialyzed with Kif18A (Fig. 7); 3) study of sustained neurotransmitter release during Kif18A action (Fig.9); 4) evaluation of an acute increase in stathmin-1 on neurotransmission (Fig. 10).

I only have a few questions:

a) The authors suggest that the application of soluble tubulin to the synapses depolymerizes the existing microtubules (Fig. 2, 3). Has the depolymerization been demonstrated? Why would it take place?

As we have explained above, we have removed all the data related to the addition of soluble tubulin. Imaging of EB3-tdTomato (Fig.1, movie S1) shows that some microtubules present in the axosomatic region are dynamic, i.e. display growth, which agrees with the staining for EB3 and synapsin I visualized by STED (Fig. 2). Together, these two sets of experiments suggest that there is a population of labile microtubules in the presynaptic terminal, which would be particularly sensitive to the action of depolymerizing agents such as SBTub3 or Kif18A. Such possibility has been validated by reconstructing four different axon terminals of a neuron dialyzed with Kif18A. The total length of the presynaptic microtubule network observed in control conditions was reduced from 11±3 μm/terminal (n=5) to 4±1 μm/terminal (lines 284-285). Although we still do not know how the addition of soluble tubulin affects the stability of presynaptic microtubules, these results show that there is a dynamic population in the presynaptic terminal.

b) In principle, the simplest interpretation of the results presented here is that soluble tubulin, either coming from depolymerized microtubules or from exogenously added tubulin, enhances synaptic release. The authors should comment on this in their discussion.

Thanks for raising this point. We did not comment on soluble tubulin and it is indeed important. The work of Honda et al. (JBC, 2002) showing that beta tubulin binds to synaptotagmin indicates that synaptic vesicles can directly interact with tubulin. We believe that the presence of a large synaptic vesicle cluster could affect microtubule growth considering that the interaction with synaptotagmin promotes polymerization. But, it is also possible that the establishment of an interaction between beta-tubulin and synaptic vesicles, i.e. synaptotagmin or synapsin, could somehow be important for neurotransmitter release. These views are presented in the discussion (lines 429-438).

c) Figure 8 is confusing. Why did the authors combine stimulation and periods of spontaneous release recordings? As the KIF18A treatment induces an increase in spontaneous release, it should result in a relatively rapid loss of EPSC amplitude also in the absence of stimulation, assuming that the same vesicles participate in spontaneous and in stimulation-evoked release. Could the authors proof this situation, by repeating the experiments from Fig. 8 in the absence of 14-AP/20Hz trains, but rather giving only single APs at rare intervals?

We have clarified this aspect of the manuscript (Figs. 8 and 9, lines 294-349). We combined the use of high-frequency trains of stimuli to deplete synaptic vesicle pools with periods without stimulation to measure spontaneous neurotransmission (Fig. 8). The intracellular solution contained 10 mM EGTA, which prevented asynchronous release. The protocol was designed to get separate measurements of evoked and spontaneous neurotransmission. Upon observing the onset of Kif18A action, which was reflected as an increase in mEPSCs, we applied bafilomycin. The faster decay in EPSC amplitude observed in neurons dialyzed with Kif18A was attributed to a more intense membrane recycling associated to the enhancement of spontaneous neurotransmitter release. If we applied single stimuli instead of trains, we might observe the effect of bafilomycin but in a longer time scale. For example, no rundown was observed in responses to single stimuli in a time window of 12-30 min min (Figs. 5c and Fig. 6d). The experiments shown in Fig. 8 involved the dialysis of neurons for ≥30 min. Since the number of vesicles mobilized by single action potentials would be lower, experiments would be prolonged, i.e. ≥45 min. This time scale could increase variability and drive to undesired effects such as rundown of responses.
Based on the comment of this reviewer we deemed appropriate to investigate the relationship between evoked and spontaneous neurotransmission upon microtubule depolymerization (Fig. 9). We confirmed that vesicles participating in the two types of neurotransmitter release were drawn from a common pool. The increase in mEPSC frequency observed in neurons dialyzed with Kif18A impaired the ability of neurons to mobilize synaptic vesicles for evoked neurotransmission, which was evidenced by a gradual decay of the readily releasable pool (RRP) and the rate of sustained neurotransmitter release. The effect on evoked neurotransmission can be a consequence of a depletion of synaptic vesicles, an impairment of synaptic vesicle supply, or a combination of both.

Reviewer #2 (Remarks to the Author): Review of Valesco et al.
We thank the reviewer for their time and careful consideration of our work.

This is an interesting manuscript focused on how microtubules in presynaptic endings affect synaptic vesicle release in autaptic synapses of superior cervical ganglion single-cell microcultures. However, even though the authors conduct solid electrophysiology there is little mechanistic insight gained and many parts of the manuscript are overinterpreted. A strength of the paper is that they use relatively newly developed mechanism to depolymerize microtubules with the photochemical SBTub3, that can rapidly depolymerize microtubules upon exposure of blue light, in conjunction with whole cell recording of EPSCs/mEPSCs. They combine these experiments with fixed cell STED imaging, EM tomography and dialysis of tubulin and a depolymerizing motor protein in the recording pipet, which allows them to clearly probe the association of microtubules and synaptic vesicle release.
Our original paper evaluated how neurotransmission was affected by an acute increase in the number of microtubules as well as by the synchronous depolymerization of microtubules. We found that addressing all reviewers' comments would significantly increase the length of the manuscript, which already contained 8 figures. Since most of the criticisms were related to microtubule depolymerization, we decided to exclusively focus on this aspect to elaborate a clearer, more specific work. This new version of the paper contains 10 figures. We have done the following four new sets of experiments: 1) live imaging of EB3-tdTomato comets (Fig. 1); 2) automated serialEM of a neuron dialyzed with Kif18A (Fig. 7); 3) study of sustained neurotransmitter release during Kif18A action (Fig.9); 4) evaluation of an acute increase in stathmin-1 on neurotransmitter release (Fig. 10).

However, it is unclear if microtubule polymerization/depolymerization and spontaneous neurotransmitter release are directly or indirectly linked. For example, does depolymerization of microtubules result in the release of microtubule binding factors that influence spontaneous release?
This is an excellent question. We provide three pieces of evidence linking microtubule depolymerization to the enhancement of spontaneous neurotransmitter release: the actions of SBTub3 (Figs. 4 and 5), Kif18A (Figs. 6-9) and stathmin-1 (Fig. 10). It is possible that an increase in soluble tubulin or any other factor liberated by microtubule catastrophe facilitates neurotransmitter release. This might be the key to the observed effects. We have looked carefully in the literature for a possible mechanism but without success. The discussion now brings the possibility that the interaction of tubulin dimers with synaptic vesicle proteins, such as synaptotagmin (Honda et al., JBC 2002) could be important for the observed effects. We cannot, however, speculate on a specific mechanism with the information available (lines 431-438).

The authors suggest that depolymerization of microtubules do not release synaptic vesicles directly, that then fuse spontaneously, which is in line with their experimental results. However, in the discussion they suggest that microtubules may act as a brake on the synaptic vesicle cycle – how would this be accomplished?
Synchronous depolymerization of presynaptic microtubules is unlikely to occur under physiological conditions (lines 406-413), therefore the presence of a wide presynaptic microtubule network is expected (Fig. 3). The paper by Rothman et al., (eLife 2016) shows that presynaptic organelle crowding has an impact on the kinetics of neurotransmitter release, particularly during high frequency neurotransmission. Polymerized microtubules could favor a maximum organelle density, i.e. by affecting the positioning of endosomes and mitochondria

(see Figs. 3 and 7) and thus limit synaptic vesicle mobility and the access to release sites. This view is now described in lines 445-450.

Overall, this is an intriguing manuscript, but their overinterpretation of many of their results and their lack of mechanistic insight into how microtubules affect spontaneous release diminish the impact of their results. Specific comments are below.

-It would be useful to mention that these are superior cervical ganglion cultures at the beginning of the Results section and indicate that this type of neuron actually makes autaptic synapses in vivo, if that is the case. If these neurons do not normally form autaptic synapses, how is this work relevant to the normal functioning of neurons?

We now mention at the beginning of the Results section that single cell microcultures are established from rat superior cervical ganglion (SCG) neurons (lines 91-93). The capacity of SCG neurons to establish autapses was extensively described in a series of papers by Furshpan and collaborators (J. Neuroscience, 1986). These works were key to understand the plasticity of synapses established by superior cervical ganglion neurons, i.e. their ability to switch from an originally adrenergic to a cholinergic phenotype. On the bases of the work of Furshpan and collaborators, we set up SCMs in my lab (Perez-Gonzalez, J. Physiol, 2008). The key advantage to hippocampal autaptic cultures is the capacity of SCG neurons to develop in the absence of a glial feeder layer. Neuronal growth can be achieved with the addition of a high concentration of NGF. Autapses show the typical properties of cholinergic synapses established in autonomic ganglia, i.e. duration of EPSCs, postsynaptic receptor composition, etc. For example, SCMs allowed us to identify the capacity of SPARC, a glial secreted protein to eliminate synapses (López-Murcia et al., PNAS, 2015). These in vitro observations were reproduced in vivo and the impairment of synaptic stability by SPARC has been confirmed by other groups. Although the current study has all the inherent limitations of in vitro studies, we believe that this work illustrates a fundamental property. Our observations of presynaptic terminal ultrastructure show many conserved features with small presynaptic terminals present throughout the nervous system. The current paper prompts to consider the presence of a functionally relevant, labile population of microtubules in the presynaptic compartment.

The authors never show any live-cell imaging with EB3. The antibody labeling of EB3 and synapsin conducted with STED microscopy in Fig. 1 is useful but does not give a sense of dynamics of microtubules in presynaptic structures. Live cell imaging of EB3 during their manipulations would provide important information regarding the effect of their manipulations (tubulin/Kif18A dialysis and SBTub3 activation).

This is a very good point. We infected SCMs with lentiviruses to express EB3-tdTomato. Fig.1b-g show images taken at the level of the cell body of a neuron. EB3 comets appear along the axon and in the perisomatic region, which is enriched in synaptic contacts (lines 98-103, see also movie S1). This example illustrates the dynamism of microtubules in synaptic locations. Live imaging during manipulations involves patching the neuron and either photoactivating SBtub3 or waiting for Kif18A to make its action (total experimental time 15-30 min). Considering background noise and the photobleaching of EB3-tdTomato, these experiments are technically demanding. Moreover, they do not solve one of the problems of autaptic cultures, which is the impossibility of distinguishing by conventional optical microscopy between EB3-tdTomato present in the pre and the postsynaptic compartment. The use of STED microscopy complements live imaging experiments by showing that microtubule growth took place in approximately 2/3 of the axosomatic terminals analyzed. Further evidence is provided by Fig. 7, where a neuron dialyzed with Kif18A was processed for automated serialEM. The reconstructions show the shortening of microtubules, thus evidencing the sensitivity of presynaptic microtubules to depolymerization.

-The dialysis of tubulin into neurons in Fig. 2 and 3 is intriguing and the negative result with denatured tubulin is a good control. However, there are several problems with this experiment. 1) Do the authors have any proof that this technique indeed promotes microtubule polymerization, as they indicate? Live imaging with EB3 would need to be done to back up their assumption. 2) It is odd that this technique affects EPSCs but has little effect on mEPSCs. This is opposite to what is found by microtubule depolymerization with SBTub3 and dialysis with Kif18A later in the paper – these experiments only affect mEPSCs, but not EPSCs. 3) They propose that the frequency of spontaneous neurotransmitter release is unaltered, but only show two traces (Fig. 3d). Whereas they show the population quantification of mEPSC amplitudes in Fig. 3e. 4) In lines 142-146 they indicate that "…the presynaptic compartment remained largely inaccessible to newly polymerized tubulin…." How can they assume this? They subsequently indicate "…neurons can rapidly potentiate neurotransmission by synthesizing new tubulin monomers…." This is just conjecture/overinterpretation of their results.

All these points are interesting, and we acknowledge them. We agree that to complement all the part related to microtubule polymerization we must visualize growth. The strategy would be to repeat experiments in neurons expressing EB3-tdTomato. Here, there is no need to discriminate for synaptic compartments, just wait for the presence of more comets. It is necessary to setup the quantification of comet mobility and ideally obtain a concentration dependent effect. Undertaking this set of experiments requires a significant extension of the time required to elaborate a point-by-point reply and would also increase the final number of figures to 13 or 14. The discussion would also be extended, and the outcome would be a paper of excessive length. We now believe that all the data gathered on the effects of polymerization and depolymerization can be split in two manuscripts. We have suppressed the 2 figures of our original manuscript related to microtubule polymerization to make a 10 figures paper focused on the effects microtubule depolymerization. We expect to use the arguments of this comment to certify an increase in microtubule polymerization in a future work.

-In line 215 they write that "Kif18A is not present in the cytosol of SCMs…" but do not show any data or refer to any studies showing this. Then in line 235 they write that they "demonstrate that acute depolymerization of microtubule plus ends by endogenous factors…". Do they mean exogenous factors? Presumably, KIF18A and SBTub3 are not endogenous.

Sorry for the confusion. The absence of Kif18A in the nervous system can be checked in the Allen Brain Atlas (https://portal.brain-map.org). We now refer to Kif18A as a biological factor (lines 243 and 266). Fig.10 shows the effect of stathmin-1, that is indeed an endogenous factor. It is expressed in the nervous system and its levels peak in early developmental stages. We thus differentiate between Kif18A (biological factor) and stathmin-1 (endogenous factor).

-In line 239 they title the section "Microtubules dynamically interact with synaptic vesicles". This is an overinterpretation. They only show EM-tomography reconstructions. They may conclude that microtubules associate with synaptic vesicles, but dynamic interaction cannot be assumed.

We have removed this title. It is now more descriptive: "Hallmarks of microtubule organization in a small presynaptic terminal" (line 137).

-In line 183 they write that inactive E-SBTub3 is used. Shouldn't this be active E-SBTub3?

"Inactive *E*-SBTub3" is correct: the *E*-isomer of SBTub3 is inactive, the *Z*-isomer is the active tubulin depolymeriser.

-There are several grammatical errors throughout the manuscript. It would benefit from the careful inspection by someone whose primary language is English.
The language has been reviewed by native English speakers.

Reviewer #3 (Remarks to the Author):

We thank the reviewer for their time and careful consideration of our work.

Using cholinergic autaptic culture preparation from rodent SCG, Velasco et al tested the effect of microtubule-depolymerizing reagents, whole-cell applied into cell bodies on evoked and spontaneous miniature EPSCs, and found them increase mEPSC frequency. Since one of the MT-depolymerizers accelerated the rundown of evoked EPSC amplitude in the presence of bafilomycin, authors conclude that MT depolymerization increases vesicle cycling rate. This is a challenging study asking an interesting question as to the regulatory role of MTs in presynaptic terminals. Unfortunately, however, little reliable direct evidence is provided to support the authors' conclusion.

Our original paper evaluated how neurotransmission was affected by an acute increase in the number of microtubules as well as by the synchronous depolymerization of microtubules. We found that addressing all reviewers' comments would significantly increase the length of the manuscript, which already contained 8 figures. Since most of the criticisms were related to microtubule depolymerization and, following a comment of this reviewer, we decided to exclusively focus on this aspect to elaborate a clearer, more specific work. This new version of the paper contains 10 figures. We have done the following four new sets of experiments: 1) live imaging of EB3-tdTomato comets (Fig. 1); 2) automated serialEM of a neuron dialyzed with Kif18A (Fig. 7); 3) study of Kif18A action on sustained neurotransmitter release (Fig.9); 4) evaluation of an acute increase in stathmin-1 on neurotransmitter release (Fig. 10). Please find below our point-by-point reply to the comments on our manuscript.

Main problems are as follows: (1) Kif18A has no effect on the evoked EPSC amplitude, but unlike what is written in the text (189-191), SBTub3 apparently enlarges EPSC and decreases the paired-pulse ratio (Fig 5c,d) suggestive of increased release probability, although no statistical evaluation is made for the difference from controls.

We acknowledge the apparent increase in the average values of EPSCs and paired-pulse ratio upon photoactivation of SBTub3. Although the differences were not statistically significant (we did not show the stats), we increased the number of control neurons (treated with DMSO) from n=7 to n=13. New Figs. 5c and d show that both experimental groups behave similarly. No differences were observed by one-way ANOVA analysis.

(2) In bafilomycin experiments, mEPSC amplitude is shown to decrease by ~80% (Fig 8c), which is inconsistent with Ikeda & Bekkers (2009) reporting that mEPSC size is unchanged in bafilomycin although it is wrongly quoted in the text (307).

Sorry for the misquotation. We observed a gradual decrease in mEPSC size that contrasts to the observations of Ikeda&Bekkers (2009). Differences might be due to factors inherent to synapses studied. For example, the type of neurotransmitter used, i.e. acetylcholine vs glutamate or, the presence of a larger cytoplasmic pool of vesicles, i.e. ~1000 vesicles/cholinergic autapse vs ~200 vesicles/hippocampal bouton. The concomitant decrease observed for mEPSCs and EPSCs was our main argument to support that spontaneous and evoked neurotransmitter release shared a common vesicle pool. Ikeda&Bekkers observed a decrease in mEPSC frequency, a finding that led them to consider that synaptic vesicles used for spontaneous and evoked neurotransmitter release were drawn from a common pool. These views are now shown in lines 312-317.

(3) Acceleration of the EPSC rundown in bafilomycin (Fig 8c) can arise from a reduction in the number of recycling vesicles even without "acceleration in the cycling rate" (37-38).

We agree with the reviewer that our assumption was too speculative. We have investigated in more detail the causes for the accelerated rundown in bafilomycin action. The immediate consequence of microtubule depolymerization in the presynaptic compartment is an increased mobilization of synaptic vesicles used for neurotransmitter release, which is now evidenced functionally (elevated mEPSC frequency, Fig.6) and morphologically (large number of exo-endocytic pits, Fig. 7). We also investigated if the responses to high-frequency stimulation were maintained after microtubule depolymerization with Kif18A (Fig.9). Sustained neurotransmitter release was impaired, which was interpreted as a depletion of synaptic vesicle pools. Possible causes could be an excessive exocytosis, a deficit in vesicle supply (see for example, Ananda Babu et al., J. Neurosci, 2020), or a combination both. These views are now explained in the section entitled "Depolymerization of presynaptic microtubules affects the depletion and refilling of synaptic vesicle pools" (starting in line 294).

(4) Surprisingly, no data is provided as to the number of synapses formed within a cell. Since mEPSCs are recorded from cell body, their frequency should reflect summed events from individual boutons.

We did not quantify the number of synapses contributing to the recorded responses in the SCMs evaluated. In previous works we have obtained morphological estimates, see for example Velasco and Llobet, 2020, where we established a range of 20-80 synapses. In Albrecht et al 2012, we calculated that 30 to 157 independent release sites were present in SCMs using variance-mean analysis. This figure agrees with the morphological quantification of synapses assuming that presynaptic terminals contain 1 or 2 active zones. On these bases, we consider that the number of synapses contributing to recorded responses ranged from 20 to 80.

(5) Dynamic changes of MTs (30, 39,74, 338, 339) in the terminal are not shown. Real time, or at least time-lapse imaging, is necessary.

The reviewer is right. We now show Fig.1 and movie S1. The use of EB3-tdTomato illustrates a population of dynamic microtubules in the periphery of the cell body, which is a region of high synaptic density (see for example Fig.1a).

(6) "Synchronous" MT depolymerization by SBTub3 (32) is not shown in the bouton.

We now show it by using automated serialEM to reconstruct synapses of two different neurons: one dialyzed with standard intracellular solution (Fig. 3) and one with Kif18A (Fig.7). Whilst axonal microtubules are largely unaffected by Kif18A, probably denoting their stability, the length of those present in the presynaptic compartment is reduced from 11±3 μm/terminal (n=5) to 4±1 μm/terminal (lines 284-285). This observation, together with the staining of EB3 and synapsin I visualized by STED, indicates that a population of presynaptic microtubules is labile.

(7) Postsynaptic effects of tubulin (Figs 2 and 3) seem unrelated to the main results.

We decided to remove the postsynaptic effects of tubulin in this revision. Our initial aim was to provide a comprehensive description of the consequences of tubulin polymerization and depolymerization on synaptic properties. If we maintain this objective, the paper becomes extremely long and fails to deliver a clear message. We have put our efforts in demonstrating the presence of a labile population of microtubules in the presynaptic compartment whose depolymerization can affect neurotransmitter release.

(8) It is unclear whether the photo-switchable MT depolymerizer SBTub3 is giving any merit to this study.

We rather agree with reviewer 2 that it is valuable, as the tool allows temporally precise stoppage of MT polymerization, and initiation of MT depolymerization.

(9) Kif18A (1-453) is utilized as a MT-depolymerizing tool but does not "highlight the relevance of physiological mechanism" (236) particularly since it is normally unexpressed in the bouton (214, 215).

We now use stathmin-1, which is a protein expressed in the nervous system, to reveal a possible physiological mechanism. The levels of stathmin-1 are particularly high in embryonic stages and gradually decay during development. We have simulated a transient increase in stathmin-1 by placing the recombinant protein in the internal patch solution. Stathmin-1 gradients are functionally relevant (Niethammer et al., Science, 2004) and therefore, our results suggest that the acute depolymerization of presynaptic microtubules, either by stathmin-1 or any other mechanism, could have functional consequences on neurotransmission.

(10) It is unclear whether the enhancement of mEPSC frequency upon MT depolymerization can play any physiological role.

This is an aspect described at the end of the discussion (lines 466-474). The molecular mechanisms supporting the presence of spontaneous neurotransmission during the assembly of neuronal circuits (reviewed by Andreae and Burrone, 2018) are largely unknown. Our results prompt to consider the contribution of microtubule instability. Neurons must release synaptic vesicles to trigger synapse formation (see for example Friedman et al, 2000). But it is unlikely that the massive synapse formation occurring during embryonic stages exclusively relies on synaptic vesicle exocytosis triggered by action potentials. Therefore, the contribution of spontaneous neurotransmission must be considered. Immature neurons could favor neurotransmitter release by "simply" depolymerizing presynaptic microtubules. Interestingly, the levels of stathmin-1 are maximal during embryonic development, when most synaptogenesis occurs and, decay postnatally. New Fig.10 could be illustrative of a physiological mechanism, especially considering the regulatory action of phosphorylation on stathmin-1 (Cassimeris, 2002).

Reviewers' comments:

Reviewer #1 (Remarks to the Author):

The authors have addressed well all of my comments, and thus I suggest that the manuscript be published in its current form.

Reviewer #2 (Remarks to the Author):

Re-Review of Valesco

In the revised manuscript the authors have included new figures and have decreased the amount of speculation in the results section, which improves the manuscript. They have now provided three different methodologies to depolymerize microtubules (MTs) and show similar results with each method, substantiating the result that depolymerization of MTs results in spontaneous neurotransmitter release. They suggest that shrinkage of presynaptic MTs underlies this phenomenon. However, none of the techniques that are used specifically affect only presynaptic MTs. Dialysis with Kif18A or stathmin through the patch pipet would result in MT depolymerization throughout the neuron and the photoactivation of the MT depolymerizing drug that is used (SBTub3), which is more temporally specific, also would affect the entire neuron. Thus, the physiological significance of showing MT depolymerization throughout the entire neuron results in increased mEPSCs is questionable.

One of the concerns was with the lack of imaging of microtubule (MT) dynamics in the superior cervical ganglion (SCG) neurons. The authors have provided a movie and a new Fig. 1 showing comets of MT end-binding proteins (EB3). However, it is unclear whether the comet is in the cell body (postsynaptic) or in the axon (presynaptic). The authors are aware of this limitation but indicate in the text (line 100-102) that “EB3 comets were obvious along axons”. To my eye it looks like the EB3 comets are within the cell body. How can the authors claim they are in the axon? Thus, I don’t think it is worth showing EB3 comets in/along the cell body in Fig. 1. All that this figure shows is that there are a few MTs polymerizing in these neurons. It provides no specificity to where this MT polymerization is taking place.

The authors also respond by indicating that Fig. 7 shows shortening of MTs after dialysis with Kif18A. Although they see a difference in MT length in the presynaptic terminal (line 284-85 – control 11um, Kif18A – 4um) they never measure MT length in the axon and compare it to controls, so it is not possible to suggest specific shortening of MTs in the presynaptic terminal and not in the axon (or the cell body or dendrites for that matter).

Regarding my comment about Kif18A not being present in SCGs the authors responded by suggesting the Allen Brain Atlas shows it is not in the brain (line 245). What about in SCGs at the age they are using in their cultures? Proteins are differentially regulated spatially and temporally. Expression in the brain is irrelevant.

In Figure 4 there is no quantification of frequency of mEPSCs. Only amplitude is plotted in B (a label indicating amplitude would be good in that panel as well). This was indicated in the first review but was not changed.

Line 205 – Fig. S1 should be changed to Fig. S2.
Line 234 – Fig. S2 should be changed to Fig. S3.

Reviewer #3 (Remarks to the Author):

Authors have substantially revised manuscript by adding new data, but there still are multiple problems to be solved by additional experiments, figure rearrangements and rewriting.
(1) In Fig 1, MT plus end movements in presynaptic or postsynaptic compartments are shown. These dynamics can be tested for the effect of MT depolymerizer or stabilizer.
It would be useful to show reversible effects of the photo-switchable depolymerizer.
(2) In Fig 3 and Fig 7, intra-terminal organelles in control terminals and after Kif18A loading are shown, but with slightly different color coding. To gain better focus, I'd recommend integrating these data into one Figure to clearly indicate significant changes after MT depolymerization. In this respect, Fig 7f and 7g are relevant to present conclusion, therefore better to show more clearly in larger panels, whereas other color pictures can be reduced in size and/or moved to Suppl Information.
(3) In Fig 4, if possible, reversibility of SBTub3 after switching to 510 nm should be shown. If not, SBTub3 data should better be pooled with those of Kif18A (Fig6) and stathmin (Fig 10) and shown in one Figure. If authors would like to address a physiological role of MTs in spontaneous transmitter release, taxol might be utilized to freeze MT dynamics, and test whether mEPSC frequency decreases after taxol loading
(4) In Fig 8, mEPSC amplitude is measured with time after bafilomycin application. This is not a trivial experiment and authors should utilize template-based analysis to identify mEPSCs out of background noise and compare the amplitude histograms of mEPSCs and noise. It seems unrealistic to identify 10 pA out of noise as in Fig 8c. In Fig 8b rundown of mEPSC size in bafilomycin in control should be shown since the results are different from glutamatergic synapses (see below, 314-315). In Fig 8a the time of recording after bafilomycin application is unclear. Results in Fig 8 and 9 can be integrated into one Figure.
(5) In Fig 9, extrapolated asymptot of linear regression line of cumulative EPSCs correspond to the size of RRP size (N) times the mEPSC size (q), whereas the initial EPSC size represents Npq, where p indicates release probability. Whether or not mEPSC size decreases in this stimulation protocol (20 Hz train repeated every 30s) should be shown? If q stays constant like infrequent stimulations (Fig 6), N is likely reduced by repeated stimulus trains in the presence of Kif18A. From the sample trace at 30 min, it appears that EPSC size (Npq) also ran down unlike Fig 6. Since paired pulse ratio is unchanged by MT depolymerization (Fig 5d), p is unlikely changed. The release probability should be calculated from Npq/Nq and show if it undergoes changes. Cumulative EPSC data at different periods between 15 min

and 30 min should be shown. If Fig 9b represents values sampled every 5 min be clear whether the time 0 corresponds to time 15 min in Fig 9a, or utilize the same time scale as Fig 9a to avoid confusion. If authors can show rundown of RRP size after MT depolymerization convincingly, these results are most relevant to physiological role of MTs, maintaining synaptic strength via supplying vesicles to release sites.
(6) In the present manuscript, block of MT polymerization is clearly shown to increase mEPSC frequency in SCG autaptic culture. This may reflect vesicle tethering role of MTs in the nerve terminals. However, physiological role of MTs regulating mEPSC frequency remains unknown.
(7) Following statements and wordings need reconsideration:
Line 36, 308, 318, 428: “neurotransmission” should read “spontaneous transmitter release” Right use of “neurotransmission” is restricted to transmission of “presynaptic action potential” to “postsynaptic action potential”. Spontaneous vesicular release does not play direct role in signal transfer across synapses.
39-43: These are utilized as different MT depolymerizing tools, irrespective of whether they are endogenous molecules. To address physiological roles of MT dynamics, usage of tools to block normal MT dynamics (eg taxol) would be required.
58: typo “despite the despite”.
106-108: This study does not provide evidence for this sentence.
121: This percentage is comparable to that at the calyx of Held (Ananda Babu et al, 2020).
184-186: These chemicals can be loaded in autapses like Kif18A and stathmin-1 with a similar spatio-temporal control.
195-197: PST can reversibly depolymerize MTs (Eguchi et al, 2017 J Neurosci). If it is also the case for SBTub3, reversibility of its effect can be tested for results in Fig 1 and Figure 4/5. Since any kind of presynaptic damage increases mEPSC frequency, showing reversibility should provide a good control. Are these experiments infeasible?
215-216: What is meant by “this linear growth was strongly disrupted”?
229: In Fig 5, showing data in 20 mM EGTA would be enough. Also, data in Fig 5b can be integrated into Fig5a.
249: If feasible, the effect of MT depolymerizers should be demonstrated in Fig 1.
278: some wordings like “in mEPSC frequency” is missing.
287: Histograms of MT length in control and in the presence of Kif8A should be shown.
290: Frequency of autophagosome occurrence should be compared between controls and Kif8A treated terminals.
314-315: This conclusion is not supported. The results suggest that vesicles are leaky and needs continuous acidification and ACh uptake, in contrast to glutamatergic synapses, where bafilomycin reduces mEPSC amplitude to ~80% (Takami et al, 2017 J Physiol) or without such effect (Ikeda & Bekkers, 2009).
319-320: Since total terminal membrane area including those of subcellular organelles remains the same due to vesicle recycling after massive transmitter release (Heuser & Reese, 1973, J Cell Biol), it is unlikely that vesicles newly transported from soma are recruited in transmitter release within the period of acute experiments.
327, 333, 342: After Kif18A application, there is no change in the mean amplitudes of mEPSCs (q) and

evoked EPSCs (Npq) (Fig 6d) in contrast to severe decrease in RRP size (N) estimated from cumulative EPSC analysis (Fig 9). Authors claims that p increases by 8-fold after Kif18A application, whereas paired-pulse ratio (PPR) remains unchanged (Fig 5d for SBTub3). Since Katz & Miledi (1968 J Physiol), it is widely accepted at many synapses that PPR is inversely related to p, and used as an index of p. Therefore, the authors results and conclusion are very surprising. If authors are confident, they should prove it more stringently using the variance-mean analysis based solely on binomial theorem. Wang et al (2020, J Neurosci) utilized the variance-mean analysis in parallel with cumulative EPSC analysis and obtained consistent results. In the variance-mean plot, initial slope represents q building up evoked EPSCs (not spontaneous mEPSCs).
344-349: These statements and Fig 9 data are confusing. Is the “Dialysis time” in Fig 9a and 9b same? If it is, why is “RRP size” in Fig 9a at 15 min is different from that in Fig 9b? If RRP start to decrease after 16 min, cumulative EPSC data should have been shown at different periods thereafter. Evoked EPSCs in Fig 9a after 30 min dialysis of Kif18A look highly diminished, whereas the EPSC size is unchanged after 30 min dialysis, suggesting that repetitive stimulation depleted RRP in Fig 9. If it is, does the RRP size recover after some resting period? If not, MT may play indispensable role in vesicle replenishment at this cholinergic terminal. If the decay of RRP size (Nq) is similar to that of EPSC size (Npq), it indicates no change in p contrary to what is stated in line 327.
351: The data obtained using Stathmin-1 do not seem to add anything to the present study. Although it is endogenous its application does not address any physiological role of MT dynamics. I’d recommend deleting Fig 9 or move it to Suppl.
352: wording “excluded from” sounds odd. “Exogenous” would be more appropriate.
384-388: Similar findings to (i) and (iii) are reported at glutamatergic synapse (Ref 7) that should be quoted and compared.
401-404: What is meant by “high affinity to endosomes”? Do they interact at low MT concentration? An increase in endosome after MT depolymerization seems just a consequence of increased spontaneous vesicle exocytosis followed by endocytic retrieval.
426-428: the statement “MTs are determinant of exocytosis” is hardly acceptable since MT depolymerization is shown to have no effect on evoked EPSCs.
438: This speculation is hardly acceptable.
439-441: “exponential increase of spontaneous release” cannot exclude the possible vesicle untethering from depolymerizing MTs.
464-465: EPSCs may recover after some resting period in the presence of MT-depolymerizing reagents. This can be tested.

Reviewers' comments:
Reviewer #1 (Remarks to the Author):
The authors have addressed well all of my comments, and thus I suggest that the manuscript be published in its current form

We thank the reviewer for their time and for their careful consideration of our manuscript.

Reviewer #2 (Remarks to the Author):

We thank the reviewer for their time and for their careful consideration of our manuscript.

Re-Review of Valesco

In the revised manuscript the authors have included new figures and have decreased the amount of speculation in the results section, which improves the manuscript. They have now provided three different methodologies to depolymerize microtubules (MTs) and show similar results with each method, substantiating the result that depolymerization of MTs results in spontaneous neurotransmitter release. They suggest that shrinkage of presynaptic MTs underlies this phenomenon. However, none of the techniques that are used specifically affect only presynaptic MTs. Dialysis with Kif18A or stathmin through the patch pipet would result in MT depolymerization throughout the neuron and the photoactivation of the MT depolymerizing drug that is used (SBTub3), which is more temporally specific, also would affect the entire neuron. Thus, the physiological significance of showing MT depolymerization throughout the entire neuron results in increased mEPSCs is questionable.

We are aware that our experimental maneuvers to depolymerize microtubules affected all neuronal compartments but, we are confident that the effects observed were from presynaptic origin. The main evidence comes from the lack of changes in mEPSC amplitude, since it is a parameter essentially determined by the number/density of postsynaptic receptors. New Fig.4c (following suggestions from this reviewer) and Fig. 6d show that neither SBTub3 nor Kif18A altered mEPSC size. Therefore, these two manipulations did not alter the density of postsynaptic receptors.

We also monitored the holding current throughout experimental time. This parameter indicates how leaky is the plasma membrane. A small holding current reports that the cell is healthy, and gradients are maintained. For example, Rosenmund and Stevens (1997) used this parameter to check fixation time of a neuron with glutaraldehyde. We did not see any significant alteration in the holding current throughout experimental time in the experiments shown. This aspect is now indicated in the methods section (lines 545-546). Also, we did not observe changes in excitability in neurons recorded, i.e. the appearance of spontaneous action potentials, which again indicates that the acute alteration of microtubule stability did not cause an overall disruption of neuronal function.

All our manipulations were acute. The observation of an immediate increase (within a minute) in mEPSC frequency upon SBTub3 activation (Figs. 5a,e and f), which is exclusively determined by the rate of synaptic vesicle exocytosis, indicates an action localized to synapses. The temporal control offered by photowsitchable drugs is key to this conclusion. Kif18A provides a complementary proof since this molecule is transported to microtubule plus ends to induce depolymerization. Kif18A will obviously concentrate in any microtubule plus end. Assuming microtubules terminate in axon terminals, the observation of an analogous effect to SBTub3 is a further proof that the effect is presynaptic.

The proportions of stable and labile microtubules in neurons have been a matter of discussion, see for review Baas et al (2016). Our acute manipulations unlikely affect

equally to both populations and are more effective on the dynamic population of microtubules. The results presented support that labile and stable microtubules are not homogenously distributed throughout the neuron. EB3 comets invade presynaptic terminals (Fig.2) and electron tomography shows that Kif18A decreases the length of presynaptic microtubules (Fig. 7). Following the comment of this reviewer we now show that the length of axonal microtubules is unaffected (lines 287-291 and new Fig. 7f). Altogether, these results suggest that the population of dynamic microtubules is more abundant in the presynaptic terminal than in the axon. This distribution explains the actions of SBTub3, Kif18A and stathmin and we believe it is a major finding of the current paper.

One of the concerns was with the lack of imaging of microtubule (MT) dynamics in the superior cervical ganglion (SCG) neurons. The authors have provided a movie and a new Fig. 1 showing comets of MT end-binding proteins (EB3). However, it is unclear whether the comet is in the cell body (postsynaptic) or in the axon (presynaptic). The authors are aware of this limitation but indicate in the text (line 100-102) that “EB3 comets were obvious along axons”. To my eye it looks like the EB3 comets are within the cell body. How can the authors claim they are in the axon? Thus, I don’t think it is worth showing EB3 comets in/along the cell body in Fig. 1. All that this figure shows is that there are a few MTs polymerizing in these neurons. It provides no specificity to where this MT polymerization is taking place.

This figure sustains our claims.

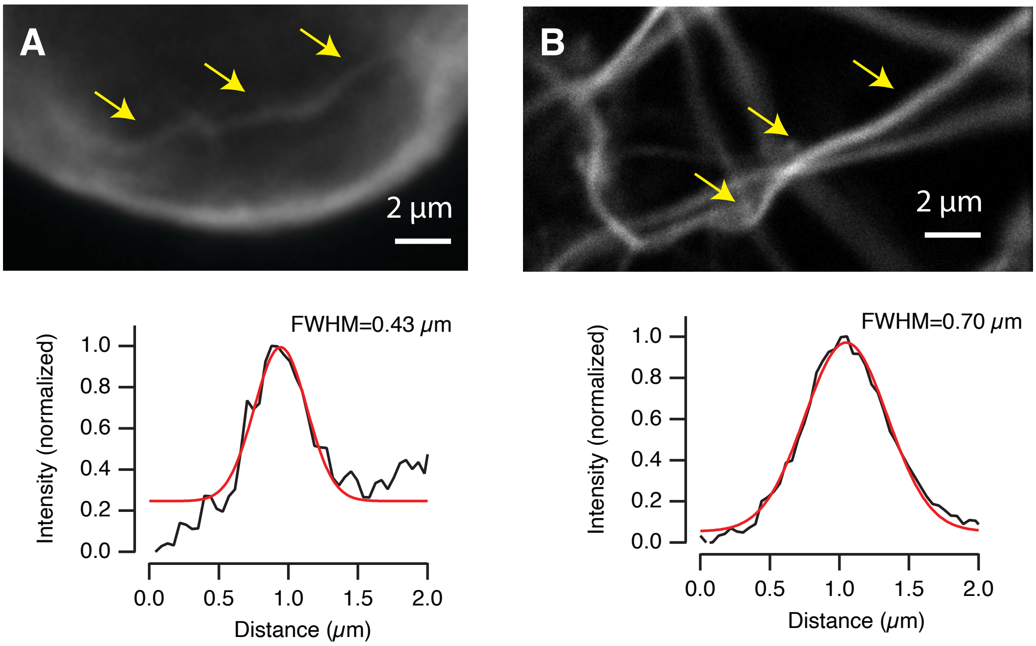

Panel A) is the average projection of all the frames shown in movie S1 , Figs. 1b-g. The arrows indicate a neuritic process that it is likely an axon due to its full width at half maximum (FWHM), which is calculated from the gaussian fit to the average intensity profile. The FWHM is 0.43 µm. Panel B) shows a region located in the periphery of the microculture, where many neurites are present. The average image

shows a neurite that contained comets (arrows). In this example the FWHM is 0.70 μm.

The FWHM of neurites containing EB3 comets ranged from 0.40 to 1 μm. We assumed panel A) was showing movement along the axon because it occurred within one of the thinnest processes observed. Since we did not apply any specific marker, we agree with this reviewer that it is not precise to term this neuronal process as an axon. We have changed the text and consider it a neurite (lines 100-101, Figure 1 legend).

We believe that the relevance of EB3-tdTomato imaging is to show the dynamics of EB3 comets in neurites. The behavior of EB3 comets, i.e. velocity, in cholinergic microcultures is similar to that described in hippocampal neuronal cultures (see for example Stepanova et al., 2010). Moreover, the apparent existence of nucleation spots is reminiscent of the findings of Qu et al (2019) if they were occurring in synapses. EB3-tdTomato imaging is thus the introduction of Fig.2, which shows that EB3 transiently invade presynaptic terminals.

The use of STED microscopy is compulsory to certify the presence of MT + ends in presynaptic terminals of autaptic neurons. Even if we were able to simultaneously image presynaptic sites and comets, i.e. visualizing synaptophysin-GFP and EB3-tdTomato, co-localization in a confocal microscope would not indicate a presynaptic location. The resolution limit established by Abbe's law does not allow to distinguish the presynaptic from the postsynaptic element in an autapse. The solution could come by doing live imaging experiments with STED. Considering the requirement of using low expression levels of EB3-tdTomato and the strong presence of bleaching, setting up this type of experiment is already a methodological project when using lasers of high power. We believe that the combination of live imaging (Fig. 1) and staining (Fig.2) are a good compromise that certify the presence of EB3 comets in about 2/3 of presynaptic terminals.

The authors also respond by indicating that Fig. 7 shows shortening of MTs after dialysis with Kif18A. Although they see a difference in MT length in the presynaptic terminal (line 284-85 – control 11um, Kif18A – 4um) they never measure MT length in the axon and compare it to controls, so it is not possible to suggest specific shortening of MTs in the presynaptic terminal and not in the axon (or the cell body or dendrites for that matter).

This is a very good point. Thanks. We now show this data in a box plot (Fig. 7f). The length of axonal microtubules in the control neuron was of 9.7 μm (n=5), which is a comparable value to the length of 12.4 μm (n=4) found in the neuron dialyzed with Kif18A. This is an important argument to support that the proportion of labile microtubules in the presynaptic compartment is higher than in the axon.

Regarding my comment about Kif18A not being present in SCGs the authors responded by suggesting the Allen Brain Atlas shows it is not in the brain (line 245). What about in SCGs at the age they are using in their cultures? Proteins are differentially regulated spatially and temporally. Expression in the brain is irrelevant.

The motor/MT depolymerizing domain of Kif18A is used as a tool to shorten microtubules. We assumed Kif18A was absent from SCG neurons but, even if the

protein were present, the dialysis of the recombinant protein into the cytosol would mimic an overexpression. This view is now reflected in the text (lines 252-254).
In Figure 4 there is no quantification of frequency of mEPSCs. Only amplitude is plotted in B (a label indicating amplitude would be good in that panel as well). This was indicated in the first review but was not changed.

Sorry about this. Data is now indicated in new Fig. 4c.

Line 205 – Fig. S1 should be changed to Fig. S2.
Line 234 – Fig. S2 should be changed to Fig. S3.

Thanks. Corrected.

Reviewer #3 (Remarks to the Author):

We thank the reviewer for their time and for their detailed comments on our manuscript.

Authors have substantially revised manuscript by adding new data, but there still are multiple problems to be solved by additional experiments, figure rearrangements and rewriting.

(1) In Fig 1, MT plus end movements in presynaptic or postsynaptic compartments are shown. These dynamics can be tested for the effect of MT depolymerizer or stabilizer. It would be useful to show reversible effects of the photo-switchable depolymerizer.

A reversion of the effects observed on spontaneous neurotransmitter release has been a long-standing yet unsuccessful, objective in this project. We sought to obtain reversible MT depolymerization using two versions of PSTs: PST-1 and PST-2 (Borowiak et al., Cell, 2015). Although both drugs increased mEPSC frequency upon illumination with 410 nm light, a consistent reversion of the effects observed was not achieved upon illumination with green light or spontaneous relaxation. In our hands, PST-1 and PST-2 were as effective as SBTub3 increasing spontaneous neurotransmitter release but, they were complicated to handle, i.e. finding a reproducible optimal concentration or light activation conditions. We never observed reversibility of their effect.

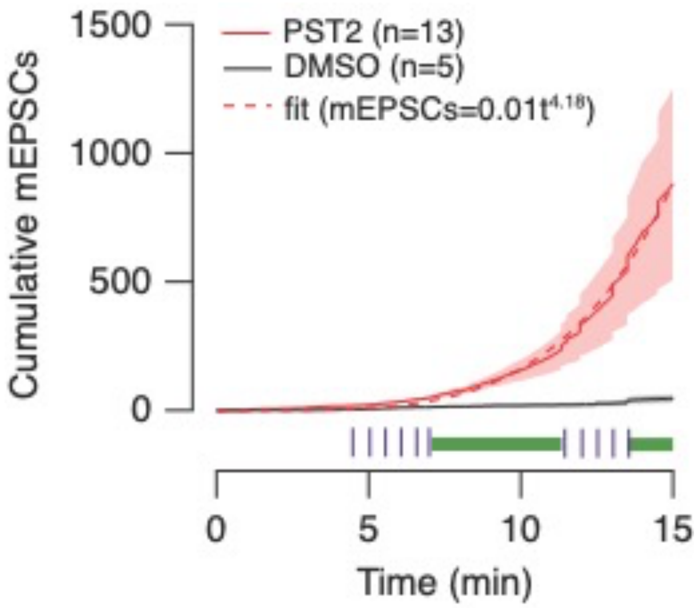

The figure shows how neurons incubated with PST-2 increased spontaneous neurotransmission upon illumination with 6 pulses of 410 nm light. Exposure to green light did not revert this effect. mEPSCs increased exponentially, thus resembling the unidirectional photoactivation of SBTub3.

We initially tested PST-1, which showed an irreversible increase of mEPSC frequency. The average spontaneous relaxation halflife of PST-1 is approximately 12 min, so we considered the possibility that green light was somehow ineffective. We

repeated experiments with PST-2, which shows a spontaneous relaxation halflife of 45 s. PST-2 should become inactive within a minute even in the dark. As shown in the Figure above, the effect of PST-2 was irreversible. We had problems finding an effective concentration of the drug and in the light of all the problems associated to PST stability (see Gao et al., 2021) we decided to use the recently developed SBTub3. This molecule does not allow reversible photoswitching but provided consistent results (Figs. 4 and 5).

We spent over a year working on the reversibility of PSTs without success. In our hands SBTubs were excellent tools because their use was straightforward. The data obtained is reminiscent of an "uncaging effect". The biggest advantage of SBTub3 is the temporal control of MT depolymerization, i.e. within seconds, which was never applied before to study neurotransmitter release.

(2) In Fig 3 and Fig 7, intra-terminal organelles in control terminals and after Kif18A loading are shown, but with slightly different color coding. To gain better focus, I'd recommend integrating these data into one Figure to clearly indicate significant changes after MT depolymerization. In this respect, Fig 7f and 7g are relevant to present conclusion, therefore better to show more clearly in larger panels, whereas other color pictures can be reduced in size and/or moved to Suppl Information.

We have updated color codes and have reduced the size of panels c-e to better illustrate Figs. 7f and g.

(3) In Fig 4, if possible, reversibility of SBTub3 after switching to 510 nm should be shown. If not, SBTub3 data should better be pooled with those of Kif18A (Fig6) and stathmin (Fig 10) and shown in one Figure. If authors would like to address a physiological role of MTs in spontaneous transmitter release, taxol might be utilized to freeze MT dynamics, and test whether mEPSC frequency decreases after taxol loading.

A key advantage of SBTub3 to photostatins (PSTs), which show reversible effects on MT dynamics upon light illumination, is robustness (see Gao et al. 2021). A disadvantage of SBTub3 is that it cannot be reverted to the E-isomer (inactive) by illumination with green light, i.e. 510 nm. This experiment is thus not feasible. In our hands, the use of SBTub3 was advantageous to find an optimal working concentration and to obtain reproducible data.

Use of taxol. Its use introduces the estabilization of MTs. Since it freezes MT dynamics it is associated with another set of MT-related cellular effects; so, we do not want to start applying an inhibitor with a different mode of action. The use of taxol implies new sets of control experiments, i.e. an evaluation of its effects on synaptic strength, paired pulse plasticity and mEPSC amplitude.

Expected effects of taxol. Our cholinergic autaptic neurons typically show a low frequency of mEPSCs (see for example Fig. 4a or Fig. 5a). The absence of mEPSCs in a period of 2-4 min is completely normal. If taxol decreases spontaneous neurotransmitter release, it will be very difficult to discriminate if a "silencing" of mEPSCs is due to taxol or if it is a behavior that can be considered normal.

If this reviewer is asking for the use of taxol to revert the effects of SBTub3 we can consider a combination of dialysis of taxol via the patch pipette with SBTub3 photoactivation. Reversibility of SBTub3 cannot be tested with this approach, since taxol concentration will gradually increase in the cytosol during experimental time.

Moreover, we cannot achieve a homogenous distribution of taxol in the cytosol so that SBTub3 would be acting on synapses with different taxol backgrounds. Using this experimental approach, we would expect a partial inhibition of SBTub3 action, i.e. less mEPSCs or a delay in their activation. Since taxol action would occur before SBTub3 effect this is not a valid approach to test reversibility.

If this reviewer is asking for the use of light-responsive taxols, they have a different set of problems as reagents - mainly related to their unavoidable lipophilicity. There are barely any recent papers using photouncaged taxol, caged taxol is no longer sold commercially and the only photoswitchable taxol has not yet been taken further in biology. So, while it is a good suggestion, we know from experience that this is more likely to generate new problems, than to deliver helpful confirmations on top of the high level of confirmation we already have by using three different MT modulators.

(4) In Fig 8, mEPSC amplitude is measured with time after bafilomycin application. This is not a trivial experiment and authors should utilize template-based analysis to identify mEPSCs out of background noise and compare the amplitude histograms of mEPSCs and noise. It seems unrealistic to identify 10 pA out of noise as in Fig 8c. In Fig 8b rundown of mEPSC size in bafilomycin in control should be shown since the results are different from glutamatergic synapses (see below, 314-315). In Fig 8a the time of recording after bafilomycin application is unclear. Results in Fig 8 and 9 can be integrated into one Figure.

The time of recording after bafilomycin application was 15 min for neurons dialyzed with internal solution. The time of recording of neurons dialyzed with Kif18A was also 15 min, however, neurotransmitter release disappeared faster than in control conditions. These times are now indicated in Fig. 8a. As shown in Figure 8c, EPSCs and mEPSCs were on average not obvious 7-8 min after bafilomycin application.

Fig. 8c now shows the average mEPSC of the recordings illustrated in the examples shown in 8a. Notice that in this example, mini amplitude decreased by ~50%. An advantage of using cholinergic synapses is the large mEPSC size, which is typically ~50 pA. We could easily track a decay in quantal size. mEPSCs below 10 pA were not resolved (noise was of 5±5 pA, mean±S.D.).

(5) In Fig 9, extrapolated asymptot of linear regression line of cumulative EPSCs correspond to the size of RRP size (N) times the mEPSC size (q), whereas the initial EPSC size represents Npq, where p indicates release probability. Whether or not mEPSC size decreases in this stimulation protocol (20 Hz train repeated every 30s) should be shown? If q stays constant like infrequent stimulations (Fig 6), N is likely reduced by repeated stimulus trains in the presence of Kif18A. From the sample trace at 30 min, it appears that EPSC size (Npq) also ran down unlike Fig 6. Since paired pulse ratio is unchanged by MT depolymerization (Fig 5d), p is unlikely changed. The release probability should be calculated from Npq/Nq and show if it undergoes changes. Cumulative EPSC data at different periods between 15 min and 30 min should be shown. If Fig 9b represents values sampled every 5 min be clear whether the time 0 corresponds to time 15 min in Fig 9a, or utilize the same time scale as Fig 9a to avoid confusion. If authors can show rundown of RRP size after MT depolymerization convincingly, these results are most relevant to physiological role of MTs, maintaining synaptic strength via supplying vesicles to release sites.

We thank the reviewer for suggesting this analysis. We followed their suggestions and now the plots of RRP size and fraction of the RRP released by the first stimulus of the train (Pr) are shown for neurons dialyzed with control internal solution (Fig. 9b) and Kif18A (Fig. 9c).

We are now showing values every 30 s, since we had these data. We wrongly believed that displaying data every 5 min provided a clearer representation.

Figs. 9b and 9c show that after 15 min of recording in the whole cell configuration, neurons dialyzed with control internal solution showed a stable RRP during 15 min. Synapses operated at a stable Pr of ~0.5-0.6. In contrast, the RRP of neurons dialyzed with Kif18A decreased by almost 40% from 15 to 30 min of experimental time with Kif18A dialysis. As observed in controls, Pr remained constant at ~0.6 throughout the period evaluated.

The analysis of Figs. 9b and 9c shows that microtubule depolymerization impairs refilling of the RRP. Data are reminiscent of results from the Takahashis's group obtained in the calyx of Held (Ananda Babu, 2020). These views are now shown in lines 350-366.

(6) In the present manuscript, block of MT polymerization is clearly shown to increase mEPSC frequency in SCG autaptic culture. This may reflect vesicle tethering role of MTs in the nerve terminals. However, physiological role of MTs regulating mEPSC frequency remains unknown.

We do not agree with this reviewer. We demonstrate using three different molecules: SBTub3, Kif18A and stathmin, that MT depolymerization has a direct impact on neurotransmitter release. This is evidence for a physiological relevance. We agree that SBTub3 and Kif18A are convenient but, artificial approaches. This is not the case of stathmin. It is a protein whose expression in the nervous system is highly regulated. In embryonic stages stathmin levels are high and gradually decay during postnatal development. Stathmin isoforms have been extensively studied and the relevance of its levels to diseases of the nervous system is state-of-the-art research (see for example Krus et al., Cell Reports, 2022, Guerra San Juan, Neuron, 2022). Our results are a clear demonstration that an abnormal increase in stathmin will likely promote an enhancement of spontaneous neurotransmitter release.

In a broader view, it is well established that the stability of microtubules increases over development. We believe that our results provide a possible explanation for the high frequency of spontaneous neurotransmitter release occurring during the assembly of neuronal circuits. This aspect is particularly evident in hippocampal neuronal cultures (see papers of the Burrone group).

In a future scenario, we would like to reversibly depolymerize only MTs present in presynaptic terminals, i.e. by developing new optical tools, and look at neurotransmission with single synapse resolution. We do not yet have the methodology to do that but, current findings support that the effort of doing such complex experiments is worthwhile.

(7) Following statements and wordings need reconsideration: Line 36, 308, 318, 428: "neurotransmission" should read "spontaneous transmitter release" Right use of "neurotransmission" is restricted to transmission of "presynaptic action potential" to "postsynaptic action potential".

We acknowledge that the term "spontaneous neurotransmission" is inaccurate.

Line 36: we do not modify "neurotransmission" since makes an overall reference to the effect of microtubules depolymerization on neurotransmitter release or neurotransmission.
Line 308: corrected
Line 318: corrected
Line 428: corrected (now line 447)
We have reviewed the use of the term "neurotransmission" throughout the manuscript.

Spontaneous vesicular release does not play direct role in signal transfer across synapses. 39-43: These are utilized as different MT depolymerizing tools, irrespective of whether they are endogenous molecules. To address physiological roles of MT dynamics, usage of tools to block normal MT dynamics (eg taxol) would be required.

We disagree with this reviewer in the use of taxol to address the physiological role of MT dynamics. Taxol disrupts microtubule dynamics by promoting stabilization.

Our approach was based in the disruption of microtubule dynamics by promoting depolymerization. We used three different molecules. SBTub3 is a photoswitchable drug that allows to control the onset of depolymerization. Kif18A is a unique kinesin that travels to microtubule plus ends to induce depolymerization. Stathmin is widely expressed in the nervous system and promotes depolymerization under tight control of phosphorylation.

The effect of microtubule stabilization on neurotransmission is a perfectly valid question but it is out of the scope of the current work. Details are explained above (remark 3).

58: typo "despite the despite".
Thanks. Corrected.

106-108: This study does not provide evidence for this sentence.

This sentence aims to provide a possible interpretation of EB3-tdTomato imaging based on existing literature. Lines 109-113 now mention the difficulties associated to imaging of autapses, which pose a limit to interpret movement of EB3-tdTomato comets. The presence of EB3 in the presynaptic compartment can only be detected by super-resolution microscopy, i.e. STED (Fig. 2).

121: This percentage is comparable to that at the calyx of Held (Ananda Babu et al, 2020).

The similarity between results obtained in both studies is now mentioned in lines 123-124.

184-186: These chemicals can be loaded in autapses like Kif18A and stathmin-1 with a similar spatio-temporal control.

We do not agree with the reviewer. Colchicine and combretastatin A-4 (CA-4) are drugs that prevent microtubule assembly. They will affect both to minus and plus ends without spatio-temporal control. Since we were dialyzing the molecules in the entire neuron, we sought to obtain an alteration that was as selective as possible of microtubule plus ends (present in presynaptic terminals).

The advantage of using SBTub3 is that it is a photoactivable CA-4. It is only active upon light illumination and exerts an action that is reminiscent of an "uncaging

effect". The key aspect of SBTub3 is thus temporal control. The advantage of using Kif18A is that it is a kinesin that moves along microtubules until reaching plus ends where it exerts its action. The advantage of using stathmin is that it is a protein that depolymerizes microtubules in neurons (for review see Cassimeris et al. Curr. Opinion in Cell Biology, 2002). Stathmin is regulated by phosphorylation and creates free tubulin concentration gradients that are key to define its physiological action (see for example Niethammer et al., Science 2004 and Zeitz, Biophysical Journal, 2014). The combination of SBTub3, Kif18A and stathmin is therefore more informative than dialysis with CA4 or colchicine.

195-197: PST can reversibly depolymerize MTs (Eguchi et al, 2017 J Neurosci). If it is also the case for SBTub3, reversibility of its effect can be tested for results in Fig 1 and Figure 4/5. Since any kind of presynaptic damage increases mEPSC frequency, showing reversibility should provide a good control. Are these experiments infeasible?

We carefully looked for presynaptic damage. The timing of illumination was key. Long illumination periods altered neurons, which was evidenced by decreases/oscillations in the holding current. Two light pulses of 3 s provided photoactivation without cell damage. We never achieved reversibility with PSTs (see reply to remark 1). In our hands reversibility with PST-1 or PST-2 is infeasible.

215-216: What is meant by "this linear growth was strongly disrupted"?
We now state that "changed to exponential". Lines 218-219.

229: In Fig 5, showing data in 20 mM EGTA would be enough.
We disagree, Bafilomycin experiments were done in the presence of 10 EGTA to minimize asynchronous release. We believe that it is necessary to show the effect of 10 EGTA and 20 mM EGTA. They are complementary experiments.

Also, data in Fig 5b can be integrated into Fig5a.
We tried to do a single figure, but data is too cluttered. We believe that showing two figures is clearer.

249: If feasible, the effect of MT depolymerizers should be demonstrated in Fig 1.
This effect is shown in Figure 5, video S3, Gao et al.,(Cell Chemical Biology, 2021).

278: some wordings like "in mEPSC frequency" is missing.
Thanks! Corrected

287: Histograms of MT length in control and in the presence of Kif8A should be shown.
Data is now shown in box plots (Fig. 7f).

290: Frequency of autophagosome occurrence should be compared between controls and Kif8A treated terminals.

Autophagosomes were not observed in any of the presynaptic terminals dialyzed with control internal solution (n=5). In contrast, were obvious in 2 terminals (n=4) dialyzed with Kif18A(1-453). Lines (296-299).

314-315: This conclusion is not supported. The results suggest that vesicles are leaky and needs continuous acidification and ACh uptake, in contrast to glutamatergic synapses, where bafilomycin reduces mEPSC amplitude to ~80% (Takami et al, 2017 J Physiol) or without such effect (Ikeda & Bekkers, 2009).

We have removed this sentence. We now state that the two systems are different and mention that cholinergic vesicles found in SCMs are leaky (lines 319-321).

319-320: Since total terminal membrane area including those of subcellular organelles remains the same due to vesicle recycling after massive transmitter release (Heuser & Reese, 1973, J Cell Biol), it is unlikely that vesicles newly transported from soma are recruited in transmitter release within the period of acute experiments.

We completely agree. There is no evidence for the contribution of synaptic vesicle precursors to massive neurotransmitter release. Since our experiments depolymerize microtubules and therefore, potentially liberate synaptic vesicle precursors, we believe that it is worth to state that bafilomycin experiments discard this remote possibility. This is a question that cell biologists might have.

327, 333, 342: After Kif18A application, there is no change in the mean amplitudes of mEPSCs (q) and evoked EPSCs (Npq) (Fig 6d) in contrast to severe decrease in RRP size (N) estimated from cumulative EPSC analysis (Fig 9). Authors claims that p increases by 8-fold after Kif18A application, whereas paired-pulse ratio (PPR) remains unchanged (Fig 5d for SBTub3). Since Katz & Miledi (1968 J Physiol), it is widely accepted at many synapses that PPR is inversely related to p, and used as an index of p. Therefore, the authors results and conclusion are very surprising. If authors are confident, they should prove it more stringently using the variance-mean analysis based solely on binomial theorem. Wang et al (2020, J Neurosci) utilized the variance-mean analysis in parallel with cumulative EPSC analysis and obtained consistent results. In the variance-mean plot, initial slope represents q building up evoked EPSCs (not spontaneous mEPSCs).

We agree on the advantages of using the mean-variance analysis. We have applied it in the past in SCMs (Albrect et al., MCN, 2012). In our hands a key limitation is stability of recordings. Holding current must be constant and minimal rundown of responses must be observed. Here we have to wait for 15 min to observe the depolymerizing action of Kif18A(1-453) to start doing experiments and there is massive spontaneous neurotransmitter release. Experimental time and noise associated to a high frequency of mEPSCs makes success is unlikely.

We took advantage of the relationship of bafilomycin inhibition to release probability described by Ikeda and Bekkers (2009). The rate (r) of bafilomycin

inhibition obtained from the exponential decay depends on release probability (p) and the frequency of stimulation:

$r=p \cdot f$

Since f is the same in neurons dialyzed with control internal solution or Kif18A(1-453), the change in p can be estimated by the relationship between rKif18A/rcont. The contribution of acetylcholine leak from synaptic vesicles should be equivalent in both experimental conditions, so it does not alter the relationship.

Fig. 8 now indicates more clearly that the stimulation paradigm (f) considered the contribution of spontaneous neurotransmitter release. Therefore, the ten-fold increase in p, reflects the contribution of changing from very few to many mEPSCs.

344-349: These statements and Fig 9 data are confusing. Is the “Dialysis time” in Fig 9a and 9b same? If it is, why is “RRP size” in Fig 9a at 15 min is different from that in Fig 9b? If RRP start to decrease after 16 min, cumulative EPSC data should have been shown at different periods thereafter. Evoked EPSCs in Fig 9a after 30 min dialysis of Kif18A look highly diminished, whereas the EPSC size is unchanged after 30 min dialysis, suggesting that repetitive stimulation depleted RRP in Fig 9. If it is, does the RRP size recover after some resting period? If not, MT may play indispensable role in vesicle replenishment at this cholinergic terminal. If the decay of RRP size (Nq) is similar to that of EPSC size (Npq), it indicates no change in p contrary to what is stated in line 327.

The analysis suggested by this reviewer on RRP size/refilling has been very useful. It is now shown in Figs. 9b and 9c. Thanks for the suggestion.

351: The data obtained using Stathmin-1 do not seem to add anything to the present study. Although it is endogenous its application does not address any physiological role of MT dynamics. I’d recommend deleting Fig 9 or move it to Suppl.

We do not agree. Stathmin is a protein widely present in the nervous system whose expression is regulated during development. The depolymerizing activity of stathmin is regulated by phosphorylation. Stathmin is a protein of interest to diseases of the nervous system and its relationship to spontaneous neurotransmitter release is a relevant information that cannot be placed in supplementary information.

352: wording “excluded from” sounds odd. “Exogenous” would be more appropriate.

We agree. We have changed this sentence. Line 371.

384-388: Similar findings to (i) and (iii) are reported at glutamatergic synapse (Ref 7) that should be quoted and compared.

We acknowledge throughout the paper the contribution of the Takahshi’s group to describe the function of MTs in presynaptic terminals. MTs mediate directed synaptic vesicle mobility (Guillaud et al, eLife 2017) and MTs contribute to maintain neurotransmitter release during high frequency stimulation Ananda Babu et al., 2020). See lines 124, 362 and 448. We now add a sentence mentioning that (i) and (iii) had already been described in the calyx of Held (lines 406-409).

The main finding of the current work is the increase in mEPSC frequency upon acute microtubule depolymerization, which has not been reported in the calyx of Held or any other synapse in the past.

401-404: What is meant by "high affinity to endosomes"? Do they interact at low MT concentration? An increase in endosome after MT depolymerization seems just a consequence of increased spontaneous vesicle exocytosis followed by endocytic retrieval.

Sorry for the lack of clarity. We have rephrased this statement (Lines 423-427).

"Presynaptic microtubules interacted with the endosomes that were distributed in the periphery of the cytoplasmic vesicle pool. Considering the key role of the endolysosomal system in the recycling of synaptic vesicles [33, 36], it is conceivable that microtubule dynamics affect to the positioning of endosomes, which could influence synaptic vesicle pools and neurotransmitter release".

426-428: the statement "MTs are determinant of exocytosis" is hardly acceptable since MT depolymerization is shown to have no effect on evoked EPSCs.

We agree that the term "exocytosis" is not precise since it can be interpreted as if MTs interfere with the SNARE complex. We have suppressed this statement.

438: This speculation is hardly acceptable.

This type of speculation was requested by reviewer 2. Our data does not allow to propose a precise mechanism linking microtubule depolymerization and neurotransmitter release. We are bringing forward some speculations based on the literature that can help the reader on possible mechanisms. It is just a speculation, so we have changed "consider" by "speculate". Line 459.

439-441: "exponential increase of spontaneous release" cannot exclude the possible vesicle untethering from depolymerizing MTs.

We exclude the possibility of SV untethering because it would be associated to the release of a finite pool of vesicles. Our EM data shows that MTs bound 1-2% of the cytoplasmic vesicles so that we should expect a plateau or, a slowdown of mEPSC frequency upon the release of >200 synaptic vesicles. The observation of an exponential increase suggests a massive mobilization and therefore our interpretation is that polymerized MTs act as a brake. We propose an action based on an effect in organelle crowding. This is a possibility defined by the group of Angus Silver (Rothman et al., eLife 2016). Lines 462-475.

464-465: EPSCs may recover after some resting period in the presence of MT-depolymerizing reagents. This can be tested.

We understand that this reviewer is asking to complete new Figs. 9b and c. We agree in the importance of observing reversibility of the effects described but, as explained above, we are facing several constraints. Here, the problem is experimental time. We should be doing experiments of more than 45 minutes. In our hands, obtaining reproducible data of reversibility in this time window is extremely complex. As this reviewer might be aware, variability increases with experimental time. We should probably try some optical approach instead of whole cell recordings, but this takes us to a new project.

Reviewers' comments:

Reviewer #2 (Remarks to the Author):

The main issue that was brought up in my second review of this paper was that all three techniques used here (KIF18A dialysis, SBTub3 activation, Stathmin dialysis) would affect microtubules (MTs) throughout the neuron, rather than only the synaptic MTs. To argue against this the authors have now measured microtubule length in the axon (how many individual microtubules were measured, where were they measured in the axon?) in 5-control buffer synapses and 4-KIF18 dialysis synapses. Previously, they had only measured MT length in the terminal and indicated MT length decreased significantly. Now they have data showing that in the axon the MT length is not significantly different in the control buffer vs. KIF18A dialysis (in fact Kif18A dialysis increases MT length slightly). Unfortunately, I do not find these data very convincing because the sample size is so small (it is based on serial electron tomography of 5 control and 4 KIF18A dialyzed synapses and adjacent axon segments) and they never show any data regarding the other two conditions (SBTub3, Stathmin). This is important because they are making the argument that all three conditions SPECIFICALLY depolymerize MTs at the terminals, because they suggest that MTs at the terminal are more dynamic than elsewhere in the neuron. I find this conclusion hard to believe, based on this very limited data set. For example, if they did the dialysis with KIF18A or stathmin or full field activation of SBTub3 they should see effects on all the polymerizing MTs throughout the neuron. Imaging with EB3 would show that MTs are polymerizing throughout the neuron, not just at the presynaptic terminal, and these treatments affect all the MTs in the neuron. They describe, in their response, other reasoning that their findings must be due to ONLY a presynaptic effect (no change in postsynaptic receptors, no change in holding current, increase in mEPSC frequency), but one cannot draw conclusions about MT behavior in the axon, dendrite or cell body based on these data. Thus, they continue to misinterpret/overinterpret their findings.

If they do not elect to do more specific, quantitative experiments on MT polymerization throughout the neuron, they should not suggest that only the MTs at the terminal are responsible for the effects on neurotransmitter release. For example, they should remove the term “presynaptic” when discussing MT dynamics after their treatments (KIF18A, SBTub3, Stathmin), since these are affecting MTs throughout the neuron (e.g. lines: 42, 274, 301, 496, 731, 760). They should also discuss the fact that a limitation of their study is that they cannot rule out that MT depolymerization in extrasynaptic regions may be affecting their measurements.

Additionally, I just noticed that in their revision and second revision they removed their important control of the denatured KIF18A (I did not notice this in the first revision or would have mentioned it in my review). They have a denatured stathmin control but no longer the KIF18A denatured control. The KIF18A denatured control should have been used in all the control experiments of KIF18A dialysis in Figures 6 and 7, but instead just the control buffer was used. Why the removal of the denatured KIF18A and why not use it in the controls in Fig. 6 and 7?

Reviewer #3 (Remarks to the Author):

This revised manuscript by Velasco et al is substantially improved. However, there still remain issues to be clarified.

In the results illustrated in Fig 9, RRP size ("N", filled symbols?) and Pr (open symbols?: They should be indicated in Fig 9 or in Fig 9 legend) estimated from the cumulative EPSC analysis are plotted in the presence or absence of Kif18A. Since the period of interest appears to be within 15-30 min, mean EPSC amplitude (1st EPSC in the train; NPr q) should be compared together with N and Pr with statistical difference evaluation between data at several points (eg, 15 min, 20 min and 27 min). In the presence of Kif 18A, N decreases, but Pr increases (perhaps by ~40% up until 27 min), thereby making EPSC size, ie NPrq, apparently constant during this period, like the results in Fig 6d. Later, however, N declines steeply with less increase in Pr, thereby reducing NPrq. In this protocol, one hidden issue is the q size (mean amplitude of mEPSC). Although q size is unchanged in the protocol in Fig 6, during recycling after massive exocytosis, if vesicular ACh refilling is incomplete, q size would be reduced and/or mEPSC frequency runs down due to empty vesicles involved. Since MT depolymerization by Kif18A reduces EPSC size to a low level 30 min after repetitive 20 Hz train application most likely because of enhanced spontaneous release, MTs normally plays a role of maintaining RRP size during high frequency neurotransmission by accelerating vesicle replenishment as previously suggested (Ref 7) as well as by restricting vesicles from spontaneous release as authors discovered in this study.

Detailed points are as follows:

36: "neurotransmission"→ spontaneous release.

41-43; 82-86: These sentences should be replaced by the above conclusion.

320: mEPSCs → mEPSC amplitude.

335-336: Confusing. ten-fold increase in "the probability of synaptic vesicle exocytosis" → "the rate of spontaneous exocytosis"

330-334: Unclear and confusing explanations. Better to delete.

406: "maintain synaptic responses" → "maintain evoked synaptic responses"

407: "Calyx of Held" → "calyx of Held".

Reviewer #2 (Remarks to the Author):

We thank the reviewer for their time and for their careful consideration of our manuscript.

The main issue that was brought up in my second review of this paper was that all three techniques used here (KIF18A dialysis, SBTub3 activation, Stathmin dialysis) would affect microtubules (MTs) throughout the neuron, rather than only the synaptic MTs. To argue against this the authors have now measured microtubule length in the axon (how many individual microtubules were measured, where were they measured in the axon?) in 5-control buffer synapses and 4-KIF18 dialysis synapses. Previously, they had only measured MT length in the terminal and indicated MT length decreased significantly. Now they have data showing that in the axon the MT length is not significantly different in the control buffer vs. KIF18A dialysis (in fact Kif18A dialysis increases MT length slightly). Unfortunately, I do not find these data very convincing because the sample size is so small (it is based on serial electron tomography of 5 control and 4 KIF18A dialyzed synapses and adjacent axon segments) and they never show any data regarding the other two conditions (SBTub3, Stathmin). This is important because they are making the argument that all three conditions SPECIFICALLY depolymerize MTs at the terminals, because they suggest that MTs at the terminal are more dynamic than elsewhere in the neuron. I find this conclusion hard to believe, based on this very limited data set. For example, if they did the dialysis with KIF18A or stathmin or full field activation of SBTub3 they should see effects on all the polymerizing MTs throughout the neuron. Imaging with EB3 would show that MTs are polymerizing throughout the neuron, not just at the presynaptic terminal, and these treatments affect all the MTs in the neuron. They describe, in their response, other reasoning that their findings must be due to ONLY a presynaptic effect (no change in postsynaptic receptors, no change in holding current, increase in mEPSC frequency), but one cannot draw conclusions about MT behavior in the axon, dendrite or cell body based on these data. Thus, they continue to misinterpret/overinterpret their findings.
If they do not elect to do more specific, quantitative experiments on MT polymerization throughout the neuron, they should not suggest that only the MTs at the terminal are responsible for the effects on neurotransmitter release. For example, they should remove the term “presynaptic” when discussing MT dynamics after their treatments (KIF18A, SBTub3, Stathmin), since these are affecting MTs throughout the neuron (e.g. lines: 42, 274, 301, 496, 731, 760). They should also discuss the fact that a limitation of their study is that they cannot rule out that MT depolymerization in extrasynaptic regions may be affecting their measurements.

We agree with this reviewer that our manipulations affect to the whole neuron and therefore accept to remove the term presynaptic in the lines indicated.
We have also included in the discussion the possible contribution of vesicles located extrasynaptically to spontaneous neurotransmitter release upon microtubule depolymerization (lines 473-474).

Additionally, I just noticed that in their revision and second revision they removed their important control of the denatured KIF18A (I did not notice this in the first revision or would have mentioned it in my review). They have a denatured stathmin control but no longer the KIF18A denatured control. The KIF18A denatured control should have

been used in all the control experiments of KIF18A dialysis in Figures 6 and 7, but instead just the control buffer was used. Why the removal of the denatured KIF18A and why not use it in the controls in Fig. 6 and 7?

We never used denatured Kif18A. The concentration-dependent effect of the kinesin led us to use the control buffer as a control of dialysis. We can anticipate a lack of effect of denatured Kif18A considering the null action of an equimolar concentration of denatured stathmin. The rationale is that stathmin and Kif18A act similarly on spontaneous neurotransmitter release. We agree with this reviewer that we could have evaluated the action of 90 nM denatured Kif18A, however, the lack of this control experiment does not invalidate the conclusions of the manuscript.

Reviewer #3 (Remarks to the Author):

We thank the reviewer for their time and for their careful consideration of our manuscript.

This revised manuscript by Velasco et al is substantially improved. However, there still remain issues to be clarified.

In the results illustrated in Fig 9, RRP size (“N”, filled symbols?) and Pr (open symbols?: They should be indicated in Fig 9 or in Fig 9 legend) estimated from the cumulative EPSC analysis are plotted in the presence or absence of Kif18A.

Sorry about this. The interpretation of the reviewer is correct. We now include this information in the figure legend.

Since the period of interest appears to be within 15-30 min, mean EPSC amplitude (1st EPSC in the train; NPr q) should be compared together with N and Pr with statistical difference evaluation between data at several points (eg, 15 min, 20 min and 27 min).

Statistical differences comparing minute 15 with minutes 22 and 27 are now shown (one-way ANOVA analysis followed by Dunnett’s multiple comparisons test).

In the presence of Kif 18A, N decreases, but Pr increases (perhaps by ~40% up until 27 min), thereby making EPSC size, ie NPrq, apparently constant during this period, like the results in Fig 6d. Later, however, N declines steeply with less increase in Pr, thereby reducing NPrq. In this protocol, one hidden issue is the q size (mean amplitude of mEPSC). Although q size is unchanged in the protocol in Fig 6, during recycling after massive exocytosis, if vesicular ACh refilling is incomplete, q size would be reduced and/or mEPSC frequency runs down due to empty vesicles involved. Since MT depolymerization by Kif18A reduces EPSC size to a low level 30 min after repetitive 20 Hz train application most likely because of enhanced spontaneous release, MTs normally plays a role of maintaining RRP size during high frequency neurotransmission by accelerating vesicle replenishment as previously suggested (Ref 7) as well as by restricting vesicles from spontaneous release as authors discovered in this study.

The dual effect of microtubules on presynaptic terminal function is now reflected in the abstract.

Detailed points are as follows:

36: “neurotransmission”→ spontaneous release.

Corrected

41-43; 82-86: These sentences should be replaced by the above conclusion.

Sentences have been replaced.

320: mEPSCs →mEPSC amplitude.

Thanks, corrected.

335-336: Confusing. ten-fold increase in “the probability of synaptic vesicle exocytosis” →“the rate of spontaneous exocytosis

Corrected

330-334: Unclear and confusing explanations. Better to delete.

This part of the paragraph has been deleted.

406: “maintain synaptic responses” → “maintain evoked synaptic responses”

Corrected

407: “Calyx of Held” → “calyx of Held”.

Corrected
